# Effectiveness of the SAFE eHealth Intervention for Women Experiencing Intimate Partner Violence and Abuse: Randomized Controlled Trial, Quantitative Process Evaluation, and Open Feasibility Study

**DOI:** 10.2196/42641

**Published:** 2023-06-27

**Authors:** Nicole E van Gelder, Suzanne A Ligthart, Karin AWL van Rosmalen-Nooijens, Judith B Prins, Sabine Oertelt-Prigione

**Affiliations:** 1 Department of Primary and Community Care Research Institute for Medical Innovation Radboud University Medical Center Nijmegen Netherlands; 2 Department of Medical Psychology Research Institute for Medical Innovation Radboud University Medical Center Nijmegen Netherlands; 3 Arbeitsgruppe 10 Sex- and Gender-sensitive Medicine Medical Faculty Ostwestfalen-Lippe University of Bielefeld Bielefeld Germany

**Keywords:** domestic violence and abuse, eHealth, feasibility, help seeking, intimate partner violence and abuse, mental health, randomized controlled trial, self-efficacy, web based, web-based intervention

## Abstract

**Background:**

Intimate partner violence and abuse (IPVA) is a pervasive societal issue that impacts many women globally. Web-based help options are becoming increasingly available and have the ability to eliminate certain barriers in help seeking for IPVA, especially in improving accessibility.

**Objective:**

This study focused on the quantitative evaluation of the SAFE eHealth intervention for women IPVA survivors.

**Methods:**

A total of 198 women who experienced IPVA participated in a randomized controlled trial and quantitative process evaluation. Participants were largely recruited on the internet and signed up through self-referral. They were allocated (blinded for the participants) to (1) the intervention group (N=99) with access to a complete version of a help website containing 4 modules on IPVA, support options, mental health, and social support, and with interactive components such as a chat, or (2) the limited-intervention control group (N=99). Data were gathered about self-efficacy, depression, anxiety, and multiple feasibility aspects. The primary outcome was self-efficacy at 6 months. The process evaluation focused on themes, such as ease of use and feeling helped. In an open feasibility study (OFS; N=170), we assessed demand, implementation, and practicality. All data for this study were collected through web-based self-report questionnaires and automatically registered web-based data such as page visits and amount of logins.

**Results:**

We found no significant difference over time between groups for self-efficacy, depression, anxiety, fear of partner, awareness, and perceived support. However, both study arms showed significantly decreased scores for anxiety and fear of partner. Most participants in both groups were satisfied, but the intervention group showed significantly higher scores for suitability and feeling helped. However, we encountered high attrition for the follow-up surveys. Furthermore, the intervention was positively evaluated on multiple feasibility aspects. The average amount of logins did not significantly differ between the study arms, but participants in the intervention arm did spend significantly more time on the website. An increase in registrations during the OFS (N=170) was identified: the mean amount of registrations per month was 13.2 during the randomized controlled trial and 56.7 during the OFS.

**Conclusions:**

Our findings did not show a significant difference in outcomes between the extensive SAFE intervention and the limited-intervention control group. It is, however, difficult to quantify the real contribution of the interactive components, as the control group also had access to a limited version of the intervention for ethical reasons. Both groups were satisfied with the intervention they received, with the intervention study arm significantly more so than the control study arm. Integrated and multilayered approaches are needed to aptly quantify the impact of web-based IPVA interventions for survivors.

**Trial Registration:**

Netherlands Trial Register NL7108 NTR7313; https://trialsearch.who.int/Trial2.aspx?TrialID=NTR7313

## Introduction

Intimate partner violence and abuse (IPVA) is a type of domestic violence and abuse (DVA) that affects many women. It consists of various types of violence between current or former partners: physical, sexual, psychological, and economic [1]. Globally, around 30% of women experience physical or sexual abuse or both from their current partner or former partner during their lifetime [2-4]. IPVA can be fatal and has many negative consequences for victims or survivors (from here on referred to as survivors), for example, mental health issues (eg, anxiety, depression, posttraumatic stress disorder), physical health issues (eg, injuries), social isolation, and financial or economic dependence [5,6]. Despite their frequent occurrences, IPVA and DVA are still taboo subjects, and survivors often struggle to disclose the violence or seek help due to shame, fear, stigma, feeling guilty, not recognizing IPVA, financial dependency, residency permit dependency, and children being involved [7-9]. Furthermore, lockdowns and mitigation measures during the COVID-19 pandemic [10] that occurred simultaneously with a part of this study not only exacerbated IPVA but also heightened the barriers for disclosing and seeking help [11-14].

While Van Rosmalen-Nooijens and colleagues [15] showed that web-based support is helpful in the context of family violence [15], the pandemic increased the urgency of web-based help being available for people facing DVA or IPVA. Thus, multiple countries, such as Australia, Italy, and Portugal, started to provide or enforce existing web-based support options [16-19]. In the Netherlands, various DVA organizations implemented and extended web-based support options as well, for example, web-based chats [20]. Indeed, web-based tools and interventions can increase accessibility of support and help options. eHealth is still relatively new in the field of IPVA research, but several studies showed the feasibility and effectiveness that web-based interventions have in supporting IPVA survivors. For example, in improving mental health, decreasing exposure to IPVA, and increasing awareness, safety behaviors, and feeling supported [21-27]. However, in a systematic review and meta-analysis, Linde and colleagues [28] found no effects on IPVA exposure, depression, or posttraumatic stress disorder when comparing eHealth interventions (including telephone and email) to standard (offline) care, control websites, or other control means, such as emails and web-based TV shows. The assessed studies did differ in type of intervention, control, and outcome measures, challenging the possibility to provide an overview about effectiveness of eHealth versus offline care [28]. Furthermore, eHealth interventions generally do not aim to replace offline support, and control websites can have an educational or supportive effect in itself.

All the aforementioned outcomes for eHealth interventions in the IPVA context originate from Australia, Canada, the United States, and New Zealand. In Europe, our team in the Netherlands developed the first eHealth intervention for female IPVA survivors that was scientifically evaluated through a randomized controlled trial (RCT), a process evaluation (PE), and an open feasibility study (OFS). The Dutch web-based intervention SAFE [29] was inspired by the Australian I-DECIDE intervention [30] and the Dutch Feel the Vibe intervention [31] and based on scientific knowledge and the insights from Dutch IPVA survivors and professionals [32]. The development process of the intervention and the study protocol for the RCT, PE, and OFS are available elsewhere [32,33]. This study focuses on 2 main outcomes derived from the RCT, quantitative PE, and OFS: effectiveness and feasibility. The primary research question is: “Is SAFE more effective in increasing self-efficacy in women exposed to IPVA than a minimal intervention?”

Secondary research questions are: “Is SAFE an effective intervention to increase awareness and perceived support and to lower symptoms of mental health problems in women exposed to IPVA?” and “Is SAFE a feasible tool in the real-world setting for providing information and support to women exposed to IPVA?”

## Methods

### Ethics Approval

All research components, including 2 amendments, covering a clarification of the inclusion criteria for fear of partner scores and the introduction of the OFS, were approved by the Medical Ethics Committee from Arnhem and Nijmegen (NL68268.091.18; dossier 2018-5009) and the RCT was registered at the Netherlands Trial Register NL7108 (NTR7313). The SAFE study was conducted in compliance with the Declaration of Helsinki, and this study is described based on the CONSORT-EHEALTH (Consolidated Standards of Reporting Trials of Electronic and Mobile HEalth Applications and onLine TeleHealth) guidelines and the CHERRIES (Checklist for Reporting Results of Internet E-surveys) [34,35]. Since the study protocol has been elaborately described and published [33], we here only briefly describe the methods, including the changes to protocol. This study consists of an RCT, PE, and OFS.

### Framework of RCT

The RCT is a parallel-group design with 2 arms and stratified (block size of 4) automated randomization in 2 age groups (18-30 years and 31-50 years). The eHealth developer and a statistician generated the random allocation sequence. The randomization was single-blinded for the participants. The researchers could track which participant was part of the control or intervention group but could not influence the randomization process. The RCT intervention group received the complete intervention, that is, access to a website with support for IPVA survivors with interactive components. The control group received minimal intervention with only the most essential static information (Textbox 1). The primary outcome to determine the intervention’s effectiveness is self-efficacy at 6 months (M6). The secondary outcomes are anxiety, depression, awareness, fear of partner, and perceived support. The outcomes were assessed with web-based self-report questionnaires at the registration process (M0), at 3 (M3), 6 (M6), and 12 months (M12; Multimedia Appendix 1; source: [33]).

The participants for the RCT were largely recruited on the internet between April 1, 2019, and October 1, 2020, and signed up through self-referral or a DVA, social, or mental health care professional. Women in the RCT were between 18 and 50 years of age who had a sufficient comprehension of Dutch, experienced IPVA no longer than 1 year ago or were still experiencing significant fear of their partner. All participants digitally received an information letter and signed an informed consent form by checking a box. Through the information letter and a statement on the intervention website, participants were made aware that this study was conducted by researchers from the Radboudumc. Subsequently, we enforced a mandatory 24-hour waiting period to ensure participants had sufficient time to contemplate their decision to participate. Participants then provided digital consent again, filled out the M0 (baseline) questionnaires, and were randomized in the control or intervention arm. The intervention was frozen during the RCT, meaning no major changes to the intervention were made during the trial.

The sample size was calculated based on the primary outcome measure, self-efficacy at 6 months, as described in the study protocol [33]. The data were analyzed with descriptive statistics (based on intention to treat), analyses of covariance (ANCOVAs), and generalized estimated equations for the primary and secondary outcomes, controlling for baseline scores, and using SPSS (version 25; IBM Corp) [36]. For self-efficacy, a complete case analysis was conducted as well for M0, M3, and M6. Due to a high dropout rate on follow-up questionnaires (198 at M0; 42 at M6), we performed an extensive check for selective attrition bias at the M6 General Self-Efficacy Scale (GSE) survey, using various variables on demographics, study arm, IPVA experiences, and the primary and secondary measures at baseline (Multimedia Appendix 2).

**SAFE modules and functionalities during randomized controlled trial (boldfaced: available to the control and intervention group; not boldfaced: only available to the intervention group) and open feasibility study (in italics: not available or applicable during OFS). OFS: open feasibility study.**

**Module—My situation:**

- **Information on intimate partner violence and abuse (IPVA).**
- **Information on unhealthy relationships.**
- **Information on the impact of IPVA on children.**

**Module—Help:**

- **Information on various help options.**
- **Information on safety (eg, in preparing to leave an abusive partner).**
- **Help database with help options, including filters for type of help and region.**

**Module—My health:**

- Information on physical health (issues).
- Information on mental health (issues).
- Information on coping strategies and stress reduction.

**Module—My environment:**

- Information on social support.
- Information on disclosing IPVA.
- Information on contact options.

**Contact:**

- **Links to contact options with fellow survivors.**
- **Option to contact the community managers.**
- *Chat, forum, diary.*

**About SAFE:**

- **Information on the SAFE research project (including patient information letter and stakeholders who provided input).**
- **Information on safety measures.**
- **Information on the community managers.**

**Additional functionalities (throughout the intervention when applicable):**

- Exercises for creating awareness and to stimulate reflecting on their situation and help seeking process.
- Short videos by women survivors of IPVA and by professionals.
- Stories and quotes from women survivors of IPVA.
- **Tips for books, films, activities, etc.**

### Framework of PE and OFS

PE consists of surveys at several time points. We also conducted a qualitative PE (interviews), described in a separate article (van Gelder NE, et al, unpublished data, 2023). The OFS tests the intervention in a real-world setting while gathering feasibility data that are combined with additional data from the RCT on specific feasibility measures: acceptability, demand, implementation, practicality, adaptation, integration, expansion, and limited-efficacy testing [37]. Data from the PE and OFS, including the Web Evaluation Questionnaire, were used to assess the feasibility measures [37] and themes, such as ease of use, understandability, and feeling helped by the intervention [38]. Furthermore, during the PE and OFS, log data on the amount of visitors, registrations, logins, time spent on the website, and viewed pages were gathered automatically through the website, the eHealth developer, and analytics tool Matomo [39]. The OFS took place between May 1, 2021, and August 1, 2021, and the data were gathered anonymously and in line with the General Data Protection Regulation (GDPR; [40]).

For the OFS, the complete version of the intervention (Textbox 1) was available without an extensive mandatory registration procedure and baseline questionnaire. Women accessed the intervention with a nickname and self-selected password. For access to the forum, women had to answer a few questions about, for example, their gender identity and age (Multimedia Appendix 3) and provide an email address in order to ensure safety for forum users. The chat feature was not offered as initially planned, given the rapid growth in users, the inability to monitor the chat 24/7 by the community managers, and the relatively low active use. Hence, we decided to remove this option, which is a change to protocol, as a preventative measure to guarantee user safety at all times.

The Web Evaluation Questionnaire data were analyzed by comparing the mean differences between the 2 RCT study arms. The log data were analyzed with descriptive statistics, and the PE data were compared to the OFS data.

## Results

### RCT Participants’ Registrations and Demographics

**Figure 1:**
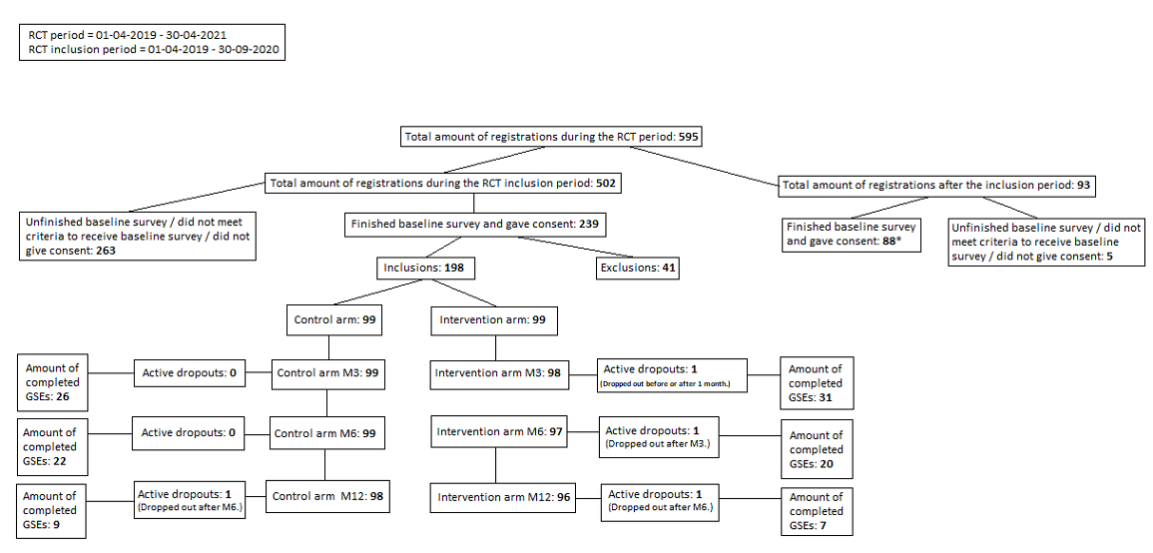
Registration flowchart during the randomized control trial (RCT) period (2019-2021). Only the group that registered within the first 12 months was followed until 12 months, and the group that registered in the additional inclusion period was followed until 6 months. GSE: General Self-Efficacy Scale. *Women who completed the baseline survey and gave consent after the inclusion period gained access to the control version of the intervention.

During the RCT inclusion period, 239 out of 502 women completed the registration they started, and 198 women were included in the RCT. Overall, 4 participants actively dropped out during their follow-up trajectory (1 from control; 3 from intervention); however, attrition on follow-up questionnaires was much higher (Figure 1).

The study sample has a mean age of 35 years. With regard to gender, all participants identify as women (answer options: “woman,” “man,” “other, namely:”, “I’d rather not say”). Most participants identify as heterosexual (179/198, 90.4%). The majority identifies (partially) as Dutch (177/198, 89.4%). More than half of the sample has a high education (100/198, 50.5%) and a paid job (115/198, 58.1%). Overall, 64.6% (128/198) of them have children and 42.9% (85/198) of them live together with their former partners. Almost all participants experienced psychological IPVA (191/198, 96.5%), and the majority experienced physical IPVA (150/198, 75.8%). Less than half reported sexual (67/198, 33.8%) or economic (86/198, 43.4%) IPVA (Table 1). A small majority (104/198, 52.5%) experienced the last IPVA incident in the week of registering for the SAFE intervention (M0), 22.2% (44/198) experienced the last incident in the month before registering, and 25.3% (50/198) experienced the last incident within the last year or longer ago.

**Table 1:** Demographics and scores of the randomized controlled trial group at baseline (M0; N=198).

Group^a^		Total (N=198)	Intervention group (N=99)	Control group (N=99)
**Age^b^ (years), n (%)**				
	18-30	26 (26.3)	26 (26.3)	26 (26.3)
	31-50	73 (73.7)	73 (73.7)	73 (73.7)
**Sexual orientation, n (%)**				
	Heterosexual	179 (90.4)	91 (91.9)	88 (88.9)
	Nonheterosexual^c^	19 (9.6)	8 (8.1)	11 (11.1)
**Country of birth, n (%)**				
	The Netherlands	170 (85.9)	83 (83.8)	87 (87.9)
	Other than the Netherlands^d^	28 (14.1)	16 (16.2)	12 (12.1)
**Cultural identification, n (%)**				
	(partially) Dutch	177 (89.4)	90 (90.9)	87 (87.9)
	Not Dutch^e^	21 (10.6)	9 (9.1)	12 (12.1)
**Religious identification, n (%)**				
	None or atheism	125 (63.1)	66 (66.7)	59 (59.6)
	Religious^f^	73 (36.9)	33 (33.3)	40 (40.4)
**Educational level^g^, n (%)**				
	Lower	98 (49.5)	53 (53.5)	45 (45.5)
	Higher	100 (50.5)	46 (46.5)	54 (54.5)
**Having children, n (%)**				
	Yes	128 (64.6)	68 (68.7)	60 (60.6)
	No	70 (35.4)	31 (31.3)	39 (39.4)
**IPVA^h^ type, n (%)**				
	Physical	150 (75.8)	80 (80.8)	70 (70.7)
	Psychological	191 (96.5)	95 (96)	96 (97)
	Sexual	67 (33.8)	34 (34.3)	33 (33.3)
	Economic	86 (43.4)	47 (47.5)	39 (39.4)
Self-efficacy (GSE^i^; range 10-40), mean		28.15	28.69	27.62
Anxiety (HADS^j^; range 0-21), mean		13.06	12.80	13.31
Depression (HADS; range 0-21), mean		9.31	8.56	10.07
Awareness (modified Contemplation Ladder; range 0-10), mean		6.59	6.65	6.54
Social support (MOS-SS5^k^; range 5-25), mean		15.85	15.82	15.88
Fear of Partner (VAS^l^; range 0-10), mean		5.82	5.97	5.68

^a^No significant differences between groups were found.

^b^The mean age of the whole study cohort was 35.3 years; intervention group, 35.5 years; control group, 35.1 years.

^c^This includes “rather not say.”

^d^Most named countries: Belgium, Colombia, Germany, Poland, South Africa, and Suriname.

^e^Participants could check multiple boxes if they identified with multiple cultures. Most named cultural identities: Belgian, Indonesian, and Surinamese.

^f^Most named religions: Christianity, Islam, and Hinduism.

^g^Lower education: primary school, secondary school, and vocational education. Higher education: higher vocational education, university, and postdoctoral.

^h^IPVA: intimate partner violence and abuse.

^i^GSE: General Self-Efficacy Scale.

^j^HADS: Hospital Anxiety and Depression Scale.

^k^MOS-SS5: Medical Outcomes Survey Social Support-5.

^l^VAS: Visual Analogue Scale.

### Effectiveness: Effect on Self-Efficacy

We could not detect any statistically significant differences in the primary outcome of self-efficacy between the study arms in the ANCOVA-M6 (*P*=.85), generalized estimated equations (*P*=.98; Table 2), and complete case analysis (*P*=.86). We noticed scores just below the general population’s average (mean 29) at M0 and just above average at M6, showing an increase in self-efficacy within 6 months for both groups. The group that scored lower on self-efficacy at M0 was significantly less likely to fill out the M6 survey (*P*=.03; mean difference >2 points, indicating real-world relevance). Women who experienced sexual IPVA completed the M6 questionnaire more often (20/42, 47.6% in follow-up group) than women who did not (47/156, 30.1% in attrition group; Multimedia Appendix 2).

There were no significant differences between the study arms on the secondary outcomes: depression, anxiety, fear of partner, awareness, social support, and perceived support by the website (Table 2). For most variables, participants in both groups showed improvement when comparing M0 to M6, with statistically significant decreasing scores for anxiety and fear of partner (*P*=.006 and *P*=.02, respectively). For depression, we found a significant difference between study arms (*P*=.03), with the intervention group scoring lower than the control group, but no significant changes between M0 and M6 in depression symptoms.

**Table 2:** Analysis of covariance (ANCOVA) and generalized estimated equations (GEE) for self-efficacy and the secondary outcomes.

Outcome measure	Range	GEE (*P* value)	ANCOVA-M0				ANCOVA-M6				*P* value
			Sample, N	Control, mean	Intervention, mean	Sample, N		Control, mean	Intervention, mean		
Self-efficacy (GSE^a^)^b^	10-40	.98	198	27.62	28.69	42		29.64	29.45	.85	
Anxiety (HADS^c^)^d^	0-21	.86	198	13.31	12.80	42		12.14	11.55	.60	
Depression (HADS)^e^	0-21	.19	198	10.07	8.56	42		10.68	8.20	.29	
Awareness (modified Contemplation Ladder)	0-10	.97	198	6.54	6.65	41		5.59	6.37	.87	
Social support (MOS-SS5)	5-25	.13	198	15.88	15.82	38		14.89	17.58	.26	
Fear of partner (VAS^f^)^d^	0-10	.46	198	5.68	5.97	42		4.73	5.30	.71	
Support by website (VAS)^g^	0-10	.88	N/A	N/A	N/A	42		4.09	5.00	.23	

^a^GSE: General Self-Efficacy Scale.

^b^No significant differences for the primary and secondary measures were found for M3 and M12. General population’s average on General Self-Efficacy Scale is 29, a higher score means a higher level of self-efficacy. No significant differences were found for self-efficacy in the complete case analysis either.

^c^HADS: Hospital Anxiety and Depression Scale.

^d^For anxiety and fear of partner, we did find significant improvements over time for both groups, *P*=.006 and *P*=.02, respectively.

^e^A significant difference (*P*=.025) was found only between the study arms, not over time, from M0 on.

^f^VAS: Visual Analogue Scale.

^g^This outcome was only measured in follow-up surveys, not at M0.

### Feasibility: Level of Need and Use

**Figure 2:**
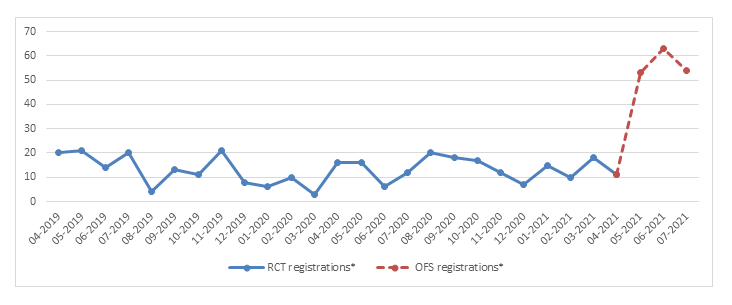
Randomized controlled trial (RCT; 2019-2021) and open feasibility study (OFS; 2021) registrations per month. For the RCT: all women who completed their registrations, regardless of inclusion or exclusion in the RCT study. For the OFS: all women who completed their registrations, anonymous (N=152) and registered accounts (N=18; mean age 36 years).

This intervention scored high on demand, implementation, and practicality. We saw an increase in registrations during the OFS (N=170; RCT and OFS means per month are 13.2 and 56.7, respectively; Figure 2). The RCT user data showed that 22 participants in the intervention group and 26 participants in the control group never logged in (Multimedia Appendix 4). The intervention group spent significantly more time on the intervention than the control group (*P*<.001), but we found no significant difference between the study arms for the average amount of logins (*P*=.08). Women in the RCT intervention group and OFS mostly visited the interactive contact options, such as the forum. The RCT control group mostly used the pages on help options (Multimedia Appendix 4). Furthermore, data on preintervention home page visits (January 2020-July 2021) show a constant flow of visitors to the SAFE website, with 2 distinctive peaks in April 2020 and July 2020. Data from SAFE’s social media accounts and mailbox show a large outreach among our target group (Multimedia Appendix 5).

Furthermore, the costs of this intervention have been higher in the development phase than in the implementation phase, and it costs relatively little to maintain (Multimedia Appendix 6). Expertise on both the technical and content sides is necessary, and thus the development, implementation, and maintenance of such an intervention need the input of multiple parties.

### Effectiveness and Feasibility: Level of Support and Appreciation

We found a significant between-group difference in feeling helped by the intervention (Table 3). The intervention group scored higher than the control group (*P*=.001) and the study arm explains this variance by 22.1% (*R*²=0.221; *P*=.001). The grade given to the intervention is significantly associated with the intervention arm as well. The intervention group (mean 7.82) was significantly more satisfied than the control group (mean 6.07; *P*<.001), with the study arm explaining 23.4% of the variance in grades between the groups (*R*²=0.234; *P*<.001). The intervention group agreed more often than the control group (*P*=.03) with the statement “SAFE suits what I want to know and what I need.” Furthermore, for both groups, the majority agrees that the language used in the intervention is comprehensible, the intervention is easy and safe to use, it suits their needs, and the speed of the website is adequate (Table 3).

**Table 3:** Web Evaluation Questionnaire outcomes for randomized controlled trial study arms.

		Intervention group, n/N (%)^a^	Control group, n/N (%)^a^
**Comprehensible language**			
	Yes	9/9 (100)	17/18 (94.4)
**Information easy to find**			
	Yes	6/9 (66.7)	8/18 (44.4)
**Provides sufficient information**			
	Yes	6/9 (66.7)	8/18 (44.4)
**Easy to use**			
	Yes	7/9 (77.8)	12/18 (66.7)
**Suits to what I want to know and what I need (*P*=.03)**			
	Yes	9/9 (100)	10/18 (55.6)
**The website is slow**			
	No	9/9 (100)	14/18 (77.8)
**Feels safe to use**			
	Yes	15/17 (88.2)	24/26 (92.3)
**Feeling helped (*P*<.001)**			
	No	1/17 (5.9)	11/27 (40.7)
	A little	6/17 (35.3)	12/27 (44.4)
	Perhaps yes	6/17 (35.5)	2/27 (7.4)
	Yes	4/17 (23.5)	2/27 (7.4)
	A lot	0/17 (0)	0/27 (0)
**Grade for intervention^b^ (*P*<.001)**			
	Not satisfied (1-5)	1/17 (5.9)	8/27 (29.6)
	Satisfied (6-7)	3/17 (17.6)	11/27 (40.7)
	Very satisfied (8-10)	13/17 (76.5)	8/27 (29.6)

^a^The N differs for the various outcomes as not all participants consistently filled out all survey questions. The percentages are rounded, and thus the total may be just under or above 100%.

^b^Mean grade for intervention was 7.82 for the intervention group and 6.07 for the control group.

## Discussion

### Principal Findings

This study quantitatively evaluated the effectiveness and feasibility of the first Dutch self-support eHealth intervention (SAFE) for women exposed to IPVA. This study did not provide statistically significant evidence that the extensive SAFE intervention was more effective than the minimal intervention in increasing self-efficacy (primary outcome), awareness, and perceived support, and in decreasing mental health problems (secondary outcomes). It did provide evidence for SAFE’s adequate feasibility on multiple levels, such as acceptability and demand, and for participants’ satisfaction and appreciation.

### Comparison to Previous Work

In line with the findings of Hegarty and colleagues [25], our study could not demonstrate a significant effect on self-efficacy of the intervention, while Ford-Gilboe and colleagues [26] did find a significant improvement over time for both study arms [26]. However, in our study and the study by Hegarty and colleagues [25], the control groups were also provided access to a limited version of the intervention, since withholding any support from women reaching out for help would have been unethical. As a consequence, the real contribution of both the overall effect of an eHealth intervention and the interactive features in particular is difficult to assess [26]. Also, since the mean self-efficacy score for both study arms was just below the general population’s average at M0, a significant change after 6 months appears unrealistic. The participants might have been facing the aftermath of ending an abusive relationship [41,42], which might have been associated with negative mental health effects for an average of at least 20 months after leaving [43]. Furthermore, the self-help nature of eHealth interventions may be more successful among people with a higher level of self-efficacy [44,45], which might not always apply to our target group.

Both groups showed significant improvements for anxiety and fear of partner but no significant effect for depression. Since anxiety was not included as an outcome in other studies, a comparison was not possible. Other studies have not found a significant impact on fear of partner [25], which might be due to structural differences in national IPVA responses. For example, Australia focused on electronically monitoring perpetrators to increase protection of survivors [46,47], while the Netherlands focused on the AWARE (Abused Women’s Active Response Emergency) system for survivors [48,49]. While other studies found significant improvements in depression over time for both study arms [25-27,50] or for a subgroup at 3 months [23], we did not find a significant effect. This might be related to external circumstances: part of this study took place during the COVID-19 pandemic, during which a global increase in depression was observed, especially in women [51-54]. Also, participants who experienced sexual IPVA were more likely to fill out the M6 questionnaire, and this type of violence can significantly exacerbate depression symptoms, even when experiencing other IPVA forms as well [55,56]. Last, the variance in outcomes for mental health problems could be explained by differences in measures, study design, and intervention design.

With regard to feasibility, the SAFE intervention scores high on acceptability, demand, implementation, practicality, adaptation, integration, and expansion. It also shows promise for certain aspects of limited efficacy testing as the intervention shows signs of being successful among the target group [37] with our large outreach in Dutch society, the amount of women that registered for SAFE, and the participants’ level of satisfaction. Our findings are similar to those from other studies, but we did find significant differences, for example, in feeling helped, in favor of the intervention study arm [23,25,26]. This finding is expected, as the intervention group received a more elaborate and interactive intervention and spent significantly more time on the website than the control group. With regard to integration [37], we noticed a possible influence of the COVID-19 pandemic and increased attention for DVA and help options during the pandemic’s first wave with an increase in registrations, especially in the first months with restrictions during a national lockdown. Also, we noticed an increase in registrations during the OFS, which was expected due to the change from an extensive registration process during the RCT to an easy, direct access registration during the OFS. Furthermore, Dutch professionals expressed their interest in web-based support and blended care for IPVA survivors [20]. Financially, the intervention’s development requires the expertise of multiple parties, and upfront development costs are relatively high, while digital maintenance—without personnel costs for monitoring of the interactive features—is limited (Multimedia Appendix 6). Taken together, the intervention’s self-help nature, its extensive reach within society (Multimedia Appendix 4), and no evidence of harmful effects make this intervention very sustainable and easy to integrate in existing care and support structures [26,37].

The SAFE eHealth intervention appears as a feasible tool to provide information and support to women who experience IPVA. Our corresponding qualitative evaluation (van Gelder NE, et al, unpublished data, 2023) shows that while the intervention did not always explicitly improve self-efficacy or mental health problems or show significant statistical differences, women did find it helpful in terms of awareness, support, and acknowledgment, and they were satisfied with the provided information and help options.

### Strengths and Limitations

First, a strength of this study is the extensive study design, using 3 evaluation methods to assess the intervention’s effectiveness on multiple levels. Second, the IPVA experiences within the study sample represent all 4 types of IPVA (psychological, physical, economic, and sexual) that survivors can endure. Third, for diversity in cultural background and sexual orientation, this study is quite representative of the general Dutch female population. In 2020, overall, 93.2% of the general Dutch female population has the Dutch nationality [57]. In this study, the majority of the women are born in the Netherlands (170/198, 85.9%) and identify (partially) as Dutch (177/198, 89.4%). For sexual orientation, in the general Dutch female population, between 1.4% and 2.4% of women identify as lesbian and 3.3% as bisexual, compared to 2.5% (5/198) of study participants identifying as lesbian and 6.1% (12/198) as bisexual [58].

There are also several limitations to this study. First, we noticed the extensive registration procedure for the RCT study was a barrier for women to sign up for the intervention. Also, unlike other studies [25-27], we encountered a high attrition rate with regard to responses on follow-up questionnaires, leading to a small sample size and possibly a power problem (eg, with social support Medical Outcomes Survey Social Support-5 [MOS-SS5]) at M6, see Table 2. With regard to attrition, we found signs of selective attrition bias for self-efficacy and sexual IPVA that may have influenced the outcomes. Furthermore, some participants might not have been able to continue their active participation due to their circumstances, priorities, mental health issues, fear of their partner finding out [59-61], or problems with executive functioning, such as processing information [62-64]. Also, part of the group who never logged in or who logged in only once may have experienced an effect from filling out the baseline survey and receiving feedback [25,26,42,65] or finding the desired information, which may have been enough to validate their experience and encourage them to seek help.

Second, the reliance on self-reports for all outcomes and thus the risk of self-reporting bias are limitations. Furthermore, as the study partially took place during the COVID-19 pandemic, this external circumstance could have decreased or delayed progress or improvement in some outcomes, for example, with regard to increasing mental health problems [52,53,66] and a rise in IPVA prevalence and severity, as well as diminished access to support services [14,20,67-72].

Third, for the measure on awareness (contemplation ladder), a lower score at M6 compared to M0 could mean that awareness became lower over time but also that they did not experience IPVA anymore or had left the abusive relationship, as scoring 0 was answering: “I don’t think of leaving my partner and/or seeking help. The relationship is not violent (anymore).” Thus, this impedes the interpretation of this outcome.

Last, in terms of diversity and equity, this study has some limitations as well [14,73]. The intervention was only available in Dutch, which excluded women who did not sufficiently comprehend Dutch. Also, while many people in the Netherlands have access to the internet (97%) and are digitally literate (50% have “above basic overall digital skills”) [74,75], women who did not have access to the internet or do not know how to use it were excluded. With regard to educational diversity, a noticeably higher percentage of the sample (50.5%) has a high education level compared to the general female population in the Netherlands (34%) [76].

### Implications

With regard to improving self-efficacy and mental health in women IPVA survivors, we could conclude that while some studies found significant improvements for both groups [26,27,50], existing eHealth interventions are generally not effective when comparing interventions to control interventions [28]. Overall, we might have to reconsider our expectations for web-based interventions since the ones specifically designed to treat symptoms of anxiety and depression only yield small effects for their target populations. Nevertheless, they can be helpful and meaningful for health outcomes in the general population [77,78]. Most importantly, IPVA survivors may not seek web-based support for this purpose. Thus, we might have to rethink how we design and evaluate these interventions. The RCT might not be the most suitable evaluation method in this context [79,80]. Instead, actively including the target group in designing the intervention and employing multiple methods of evaluation, quantitative and qualitative, appears crucial toward obtaining real-world, in-depth knowledge about the effectiveness of an eHealth intervention for IPVA survivors [32, (van Gelder NE, et al, unpublished data, 2023)]. Last, in both design and research, interventions should pay attention to diversity on multiple levels, for example, with regard to cultural sensitivity and availability in multiple languages [(van Gelder NE, et al, unpublished data, 2023) 81-83]. Currently, the SAFE intervention is available in Dutch, and the essential components have been translated into English and Arabic.

Overall, the feasibility of this intervention is high, with survivors expressing a demand for web-based options and professionals expressing interest in implementing this type of support. Hence, in addition to the direct use by IPVA survivors, professionals have the option to refer clients or patients to the platform as an additional means of support, as a bridging tool when waitlisted for an in-person intervention, or as part of a blended care approach [20,32].

## Appendix

Multimedia Appendix 1 Primary and secondary outcome measures and measurement timepoints.

Multimedia Appendix 2 Check for selective attrition bias at the M6 survey on self-efficacy.

Multimedia Appendix 3 Login prerequisites for the intervention during the OFS.

Multimedia Appendix 4 User data from the RCT study arms and OFS group.

Multimedia Appendix 5 Feasibility data from Matomo and SAFE’s social media accounts and messages.

Multimedia Appendix 6 Information on the eHealth developer costs of the SAFE intervention.

Multimedia Appendix 7 CONSORT-eHEALTH checklist (V 1.6.1).

## Abbreviations

ANCOVA: analysis of covariance
AWARE: Abused Women’s Active Response Emergency
CHERRIES: Checklist for Reporting Results of Internet E-surveys
CONSORT-EHEALTH: Consolidated Standards of Reporting Trials of Electronic and Mobile HEalth Applications and onLine TeleHealth
DVA: domestic violence and abuse
GDPR: General Data Protection Regulation
GSE: General Self-Efficacy Scale
IPVA: intimate partner violence and abuse
MOS-SS5: Medical Outcomes Survey Social Support-5
OFS: open feasibility study
PE: process evaluation
RCT: randomized controlled trial

## Data Availability

The data sets generated and analyzed during this study are available on reasonable request from the DANS repository [84].
