# Supplementary material for: Effectiveness of the SAFE eHealth Intervention for Women Experiencing Intimate Partner Violence and Abuse: Randomized Controlled Trial, Quantitative Process Evaluation, and Open Feasibility Study
Source: J Med Internet Res. 2023 Jun 27;25:e42641. doi: 10.2196/42641 (PMC10337397; doi:10.2196/42641)
Supplement: Multimedia Appendix 1 [file jmir_v25i1e42641_app1.docx]

**Multimedia Appendix 1**. Primary and secondary outcome measures and measurement timepoints.

| **Outcome** | **Measure** | **Hypothesis** | **Timepoint(s)** |
| --- | --- | --- | --- |
| ***Self-efficacy*** | **General Self-Efficacy Scale (GSE) *– Primary outcome***  Range: 10-40. General population’s average = 29. A higher score = a higher level of self-efficacy. [1-3] | The intervention group has a higher mean score at M6 than the control group. | M0, M3, M6, M12^a^ |
| ***Anxiety and depression*** | **Hospital Anxiety and Depression Scale (HADS) *– Secondary outcome***  Range: 0-21. A lower score = less symptoms of anxiety or depression. [4-6] | The intervention group has lower mean scores on anxiety and depression at M6 than the control group. | M0, M3, M6, M12^a^ |
| ***Awareness*** | **Contemplation Ladder *– Secondary outcome***  Range: 0-10. A higher score = a higher level of awareness^c^. [7] | The intervention group has a higher mean score at M6 than the control group. | M0, M3, M6, M12^a^ |
| ***Perceived social support*** | Medical Outcomes Survey – Social Support (MOS-SS5) *– Secondary outcome*  Range: 5-25. A higher score = a higher level of perceived support. [8-9] | The intervention group has a higher mean at M6 score than the control group. | M0, M3, M6, M12^a^ |
| ***Fear of partner*** | Visual Analogue Scale (VAS) *– Secondary outcome*  Range: 1-10. A lower score = a lower level of fear of partner. | The intervention group has an overall lower mean score than the control group. | M0, M3, M6, M12^a^ |
| ***Perceived support by website*** | Visual Analogue Scale (VAS) *– Secondary outcome*  Range: 1-10. A higher score = a higher level of perceived support. | The intervention group has an overall higher mean score than the control group. | M3, M6, M12^a^ |
| ***Users’ evaluation of the intervention*** | Web Evaluation Questionnaire (WEQ) | The intervention group has an overall higher mean score than the control group. | M1, M3, M6^b^ |
| ***Demographics, IPVA*** | General Characteristics Questionnaire (GCQ) | N/A | M0, M3, M6, M12^a^ |

*Note*: participants received a maximum of two e-mail reminders if they did not complete a questionnaire timepoint. | ^a^M12: Only participants who registered between 1 April 2019 and 1 April 2020 received follow-up questionnaires at 12 months. Participants who registered after this period received the last follow-up questionnaire at 6 months (M6). | ^b^The WEQ was filled out one month after the first login (M1), at three and six months (M3 + M6). Sending the WEQ at M3 is a protocol deviation, we decided to add this timepoint to increase the opportunity of gathering valuable user feedback. | ^c^A participant could also score lower because they left the abusive partner or the violence stopped, for example 0 = ‘I don’t think about leaving my (ex-)partner and / or seeking help. The relationship is not abusive (anymore).’

**References**

**General Self-Efficacy Scale (GSE)**

1. Rensen C, Bandyopadhyay S, Gopal PK, Van Brakel WH. Measuring leprosy-related stigma–a pilot study to validate a toolkit of instruments. Disability and Rehabilitation. 2011;33(9):711-719. PMID: 20690861
2. Schwarzer R, Jerusalem M. The general self-efficacy scale (GSE). Anxiety, Stress, and Coping. 2010;12:329-345.
3. Teeuw B, Schwarzer R, Jerusalem M. Dutch adaptation of the general perceived self-efficacy scale. See: http://userpage.fu-berlin.de/~ health/dutch.htm; 1994.

**Hospital Anxiety and Depression Scale (HADS)**

1. Bjelland I, Dahl AA, Haug TT, Neckelmann D. The validity of the Hospital Anxiety and Depression Scale: an updated literature review. Journal of psychosomatic research. 2002;52(2):69-77. PMID: 11832252
2. Spinhoven P, Ormel J, Sloekers P, Kempen G, Speckens A, Van Hemert A. A validation study of the Hospital Anxiety and Depression Scale (HADS) in different groups of Dutch subjects. Psychological medicine. 1997;27(02):363-370. PMID: 9089829
3. Zigmond AS, Snaith RP. The hospital anxiety and depression scale. Acta psychiatrica scandinavica. 1983;67(6):361-370. PMID: 6880820

**Contemplation Ladder**

1. Biener L, Abrams DB. The contemplation ladder: validation of a measure of readiness to consider smoking cessation. Health Psychol. 1991;10. PMID: 1935872

**Medical Outcomes Survey – Social Support (MOS-SS5)**

1. McCarrier K, Bushnell D, Martin M, Paczkowski R, Nelson D, Buesching D. PRM16 validation and psychometric evaluation of a 5-item measure of perceived social support. Value in Health. 2011;14(3):A148. doi:10.1016/j.jval.2011.02.824
2. Sherbourne CD, Stewart AL. The MOS social support survey. Social science & medicine. 1991;32(6):705-714. PMID: 2035047
