# Supplementary material for: Effectiveness of the SAFE eHealth Intervention for Women Experiencing Intimate Partner Violence and Abuse: Randomized Controlled Trial, Quantitative Process Evaluation, and Open Feasibility Study
Source: J Med Internet Res. 2023 Jun 27;25:e42641. doi: 10.2196/42641 (PMC10337397; doi:10.2196/42641)
Supplement: Multimedia Appendix 2 [file jmir_v25i1e42641_app2.docx]

**Multimedia Appendix 2.** Check for selective attrition bias at the M6 survey on self-efficacy.

| **Measured at M0 (baseline)** | **Follow-up group (N=42)** | **Attrition group (N=156)** | **P-value** |
| --- | --- | --- | --- |
| ***Self-efficacy (GSE)^a^*** | M=30.05 | M=27.64 | p=.028* |
| ***Anxiety (HADS)^a^*** | M=13.38 | M=12.97 | p=.570 |
| ***Depression (HADS)^a^*** | M=9.52 | M=9.26 | p=.276 |
| ***Fear of partner (VAS)^a^*** | M=6.02 | M=5.77 | p=.575 |
| ***Motivation ladder^a^*** | M=6.88 | M=6.51 | p=.544 |
| ***Social support (MOS-SS5)^a^*** | M=16.07 | M=15.79 | p=.735 |
| ***Gender characteristics (BSRI)^a^*** | M=2.38 | M=2.52 | p=.362 |
| ***Age^a^*** | M=37.88 | M=34.58 | p=.023* |
| ***Sexual orientation^a^*** | M=1.29 | M=1.15 | p=.166 |
| ***Last IPVA incident^a^*** | M=3.67 | M=3.53 | p=.522 |
| ***Study arm^b^*** |  |  | p=.728 |
|  | Control group = 52,4% | Control group = 49,4% |  |
|  | Intervention group = 47,6% | Intervention group = 50,6% |  |
| ***Children (yes/no)^b^*** |  | No = 35,9% | p=.758 |
|  | Yes = 66,7% | Yes = 64,1% |  |
|  | No = 33,3% | No = 35,9% |  |
| ***Education level (high/low)^b^*** |  |  | p=.188 |
|  | High = 59,5% | High = 48,1% |  |
|  | Low = 40,5% | Low = 51,9% |  |
| ***Living together with or without (ex-)partner^b^*** |  |  | p=.717 |
|  | With (ex-)partner = 40,5% | With (ex-)partner = 43,6% |  |
|  | Without (ex-)partner = 59,5% | Without (ex-)partner = 56,4% |  |
| ***Country of birth is the Netherlands (yes/no)^b^*** |  |  | p=.639 |
|  | Yes = 88,1% | Yes = 85,3% |  |
|  | No = 11,9% | No = 14,7% |  |
| ***Cultural identification is (partially) Dutch (yes/no)^b^*** |  |  | p=.807 |
|  | Yes = 90,5% | Yes = 91,7% |  |
|  | No = 9,5% | No = 8,3% |  |
| ***Physical IPVA (yes/no)^b^*** |  |  | p=.121 |
|  | Yes = 66,7% | Yes = 78,2% |  |
|  | No = 33,3% | No = 21,8% |  |
| ***Psychological IPVA (yes/no)^b^*** |  |  | p=.162 |
|  | Yes = 100% | Yes = 95,5% |  |
|  | No = 0% | No = 4,5% |  |
| ***Sexual IPVA (yes/no)^b^*** |  |  | p=.033* |
|  | Yes = 47,6% | Yes = 30,1% |  |
|  | No = 52,4% | No = 69,9% |  |
| ***Economic IPVA (yes/no)^b^*** |  |  | p=.932 |
|  | Yes = 42,9% | Yes = 43,6% |  |
|  | No = 57,1% | No = 56,4% |  |

*p<.05. | ^a^T-tests. | ^b^Chi-square tests.
