# Supplementary material for: Effectiveness of the SAFE eHealth Intervention for Women Experiencing Intimate Partner Violence and Abuse: Randomized Controlled Trial, Quantitative Process Evaluation, and Open Feasibility Study
Source: J Med Internet Res. 2023 Jun 27;25:e42641. doi: 10.2196/42641 (PMC10337397; doi:10.2196/42641)
Supplement: Multimedia Appendix 3 [file jmir_v25i1e42641_app3.docx]

**Multimedia Appendix 3.** Login prerequisites for the intervention during the OFS.

| **Anonymous account** | **Registered account (for access to the forum)** |
| --- | --- |
| - Nickname | - Nickname |
| - Password | - Password |
|  | - E-mail address |
|  | - Gender identity (only women can gain access)_a_ |
|  | - Age (only 18+ years old can gain access) |
|  | - Do you have personal experience with an unsafe relationship, a relationship in which you were not free, or intimate partner violence and abuse?^b^ |

^a^Answer options: woman, man, other. | ^b^Additional information that was provided for this question: ‘Intimate partner violence and abuse is every type of violence between (ex-)partners. Types of violence and abuse: emotional / psychological (e.g. humiliation, manipulation, threats, stalking); physical (e.g. hitting, kicking, shoving); financially / economically (e.g. not being allowed to work or study or to have your own bank card, partner withholding your wages); sexually (e.g. forced to perform sexual acts, rape, sextortion, online sexual abuse).
