# Supplementary material for: Effectiveness of the SAFE eHealth Intervention for Women Experiencing Intimate Partner Violence and Abuse: Randomized Controlled Trial, Quantitative Process Evaluation, and Open Feasibility Study
Source: J Med Internet Res. 2023 Jun 27;25:e42641. doi: 10.2196/42641 (PMC10337397; doi:10.2196/42641)
Supplement: Multimedia Appendix 4 [file jmir_v25i1e42641_app4.docx]

**Multimedia Appendix 4.** User data from the RCT study arms and OFS group.

| **Intervention group RCT (N=99)** |
| --- |
| ***Top 5 visited pages^a^:*** |
| 1. **Forum** (AoV^a^=601 \| UV^a^=27) |
| 1. **Chat** (AoV=308 \| UV=36) |
| 1. **Contact (module: My environment)** (AoV=161 \| UV=33) |
| 1. **Seek help (module: Help)** (AoV=136 \| UV=31) |
| 1. **Contact (Contact homepage)** (AoV=134 \| UV=31) |
| **- Minutes spent on intervention in total:** mean = 44,4 \| range = 0 – 459 |
| - **Amount of logins:** mean = 3,3 \| range: 0 – 44 |
| **- Logged in at least once:** N=77 |
| **- Never logged in:** N=22 |
|  |
| **Control group RCT (N=99)** |
| ***Top 5 visited pages^a^:*** |
| 1. **Seek help (module: Help)** (AoV=211 \| UV=47) |
| 1. **Help (homepage of Help module)** (AoV=102 \| UV=50) |
| 1. **News, books and more** (AoV=84 \| UV=26) |
| 1. **Start here (introduction page)** (AoV=80 \| UV=45) |
| 1. **Contact (Contact homepage)** (AoV=28 \| UV=15) |
| **- Minutes spent on intervention in total:** mean = 13,3 \| range = 0 – 124 |
| **- Amount of logins:** mean = 2,1 \| range: 0 – 13 |
| **- Logged in at least once:** N=73 |
| **- Never logged in:** N=26 |
|  |
| **OFS (N=170)** |
| ***Top 5 visited pages^a^:*** |
| 1. **Forum** (AoV=429 \| UV=80)^b^ |
| 1. **Seek help (module: Help)** (AoV=190 \| UV=71) |
| 1. **Types of help (module: Help)** (AoV=120 \| UV=111) |
| 1. **What is IPVA? (module: My situation)** (AoV=100 \| UV=97) |
| 1. **Types of violence (module: My situation)** (AoV=88 \| UV=85) |
| **- Minutes spent on intervention per visit:** mean = 16,1 \| range = 1 – 155 |
| **- Amount of logins:** mean = 1,26 \| range: 1 – 4 |
| **- Logged in at least once:** N=170 |
| **- Never logged in:** N=0 |

^a^The overview page that appears when logging in is not taken into account here. | AoV = total amount of visits. | UV = total amount of unique visitors. | ^b^Part of the unique visitors registered to obtain access to the forum (N=18), others visited the introduction page but did not obtain access to the forum (N=62).
