# Supplementary material for: Effectiveness of the SAFE eHealth Intervention for Women Experiencing Intimate Partner Violence and Abuse: Randomized Controlled Trial, Quantitative Process Evaluation, and Open Feasibility Study
Source: J Med Internet Res. 2023 Jun 27;25:e42641. doi: 10.2196/42641 (PMC10337397; doi:10.2196/42641)
Supplement: Multimedia Appendix 5 [file jmir_v25i1e42641_app5.docx]

**Multimedia Appendix 5.** Feasibility data from Matomo and SAFE’s social media accounts and messages.

| **Matomo during OFS (01-05-2021 – 31-07-2021)** | |
| --- | --- |
| ***Mean time spent on pre-intervention homepage:*** | 1,37 minutes |
| ***Mean amount of actions on pre-intervention homepage:*** | 3 |
| ***Mean bounce rate on pre-intervention homepage:*** | 49,7% |
|  |  |
| ***Top 3 devices:*** |  |
|  | 1) Smartphone |
|  | 2) Desktop |
|  | 3) Phablet (combination of smartphone and tablet) |
| ***Top 3 referring sources:*** |  |
|  | 1) Direct entry |
|  | 2) Via websites |
|  | 3) Via search engines |
| ***Top 3 referring websites:*** |  |
|  | 1) www.slachtofferwijzer.nl |
|  | 2) www.huiselijkgeweld.nl |
|  | 3) www.hulpapp.nl |
| ***Amount of unique clicks on outgoing links on pre-intervention homepage targeted at (1) male IPVA survivors and at (2 + 3) IPVA perpetrators:*** |  |
|  | 1) www.mannenmishandeling.nl: **6** |
|  | 2) www.deplegerinons.nl (now known as ‘De Pion’): **7** |
|  | 3) www.dewaagnederland.nl/clienten-en-familie/behandeling-volwassenen/: **2** |
|  | |
| **Social media accounts (various time periods)** | |
| ***Twitter* *(07-01-2019 – 31-07-2021)*** | Amount of followers: **754** |
|  |  |
| ***Instagram (13-08-2019 – 31-07-2021)*** | Amount of followers: **1462** |
| ***Instagram (02-06-2021 – 31-07-2021)*** | Female-male ratio: **84,4% - 15,6%** |
|  | Largest age group: **25 - 34 (31%)** |
|  |  |
| ***Facebook (12-11-2018 – 31-07-2021*** |  |
|  | **476** people follow SAFE on Facebook |
|  | Female-male ratio: **93% - 5%** |
|  | Largest age group: **35 - 44 (30%)** |
|  |  |
|  | **385** people like SAFE on Facebook |
|  | Female-male ratio: **94% - 5%** |
|  | Largest age group: **35 - 44 (29%) and 45 - 54 (29%)** |
|  | |
| **SAFE mailbox, online contact form, and PMs^a^ (01-04-2019 – 31-07-2021)** | |
| Amount of requests for help from survivors (non-registered and registered for SAFE): **60** | |
| Amount of requests for help from bystanders (e.g. family members, friends, neighbors, colleagues): **19** | |
| Amount of requests from professionals for more information about SAFE: **72** | |
| Amount of requests for help from male survivors and from bystanders about a male survivor, and amount of questions on why SAFE is only available for female survivors: **11** | |

^a^Messages received via the SAFE social media accounts were not taken into account here. People could send messages to the mailbox and via the online contact form without having to be registered for the SAFE intervention. The PM (personal message) option was available for every woman that registered with SAFE.
