# Supplementary material for: Effectiveness of the SAFE eHealth Intervention for Women Experiencing Intimate Partner Violence and Abuse: Randomized Controlled Trial, Quantitative Process Evaluation, and Open Feasibility Study
Source: J Med Internet Res. 2023 Jun 27;25:e42641. doi: 10.2196/42641 (PMC10337397; doi:10.2196/42641)
Supplement: Multimedia Appendix 6 [file jmir_v25i1e42641_app6.docx]

**Multimedia Appendix 6.** Information on the eHealth developer costs of the SAFE intervention.

| **Development (one-time costs)** | **Maintenance (yearly costs)** |
| --- | --- |
| €34.100 | €2.580 |
