# Supplementary material for: Effectiveness of the SAFE eHealth Intervention for Women Experiencing Intimate Partner Violence and Abuse: Randomized Controlled Trial, Quantitative Process Evaluation, and Open Feasibility Study
Source: J Med Internet Res. 2023 Jun 27;25:e42641. doi: 10.2196/42641 (PMC10337397; doi:10.2196/42641)
Supplement: Multimedia Appendix 7 [file jmir_v25i1e42641_app7.pdf]

CONSORT-EHEALTH (V 1.6.1) - Submission/Publication Form

The CONSORT-EHEALTH checklist is intended for authors of randomized trials evaluating web-based and Internet-based applications/interventions, including mobile interventions, electronic games (incl multiplayer games), social media, certain telehealth applications, and other interactive and/or networked electronic applications. Some of the items (e.g. all subitems under item 5 - description of the intervention) may also be applicable for other study designs.

The goal of the CONSORT EHEALTH checklist and guideline is to be
a) a guide for reporting for authors of RCTs,
b) to form a basis for appraisal of an ehealth trial (in terms of validity)

CONSORT-EHEALTH items/subitems are MANDATORY reporting items for studies published in the Journal of Medical Internet Research and other journals / scientific societies endorsing the checklist.

Items numbered 1., 2., 3., 4a., 4b etc are original CONSORT or CONSORT-NPT (non-pharmacologic treatment) items.
Items with Roman numerals (i., ii, iii, iv etc.) are CONSORT-EHEALTH extensions/clarifications.

As the CONSORT-EHEALTH checklist is still considered in a formative stage, we would ask that you also RATE ON A SCALE OF 1-5 how important/useful you feel each item is FOR THE PURPOSE OF THE CHECKLIST and reporting guideline (optional).

Mandatory reporting items are marked with a red *.
In the textboxes, either copy & paste the relevant sections from your manuscript into this form - please include any quotes from your manuscript in QUOTATION MARKS,
or answer directly by providing additional information not in the manuscript, or elaborating on why the item was not relevant for this study.

YOUR ANSWERS WILL BE PUBLISHED AS A SUPPLEMENTARY FILE TO YOUR PUBLICATION IN JMIR AND ARE CONSIDERED PART OF YOUR PUBLICATION (IF ACCEPTED).
Please fill in these questions diligently. Information will not be copyedited, so please use proper spelling and grammar, use correct capitalization, and avoid abbreviations.

DO NOT FORGET TO SAVE AS PDF _AND_ CLICK THE SUBMIT BUTTON SO YOUR ANSWERS ARE IN OUR DATABASE !!!

Citation Suggestion (if you append the pdf as Appendix we suggest to cite this paper in the caption):
Eysenbach G, CONSORT-EHEALTH Group
CONSORT-EHEALTH: Improving and Standardizing Evaluation Reports of Web-based and Mobile Health Interventions
J Med Internet Res 2011;13(4):e126

URL: http://www.jmir.org/2011/4/e126/
doi: 10.2196/jmir.1923
PMID: 22209829

Log in bij Google om je voortgang op te slaan. Meer informatie

*Vereist

Your name *

First Last

Nicole van Gelder

Primary Affiliation (short), City, Country *

University of Toronto, Toronto, Canada

Radboudumc, Nijmegen, The Netherlands

Your e-mail address *

abc@gmail.com

nicole.vangelder@radboudumc.nl

Title of your manuscript *

Provide the (draft) title of your manuscript.

Effectiveness of the SAFE eHealth intervention for women experiencing intimate partner violence and abuse: randomized controlled trial, quantitative process evaluation, and open feasibility study.

Name of your App/Software/Intervention *

If there is a short and a long/alternate name, write the short name first and add the long name in brackets.

SAFE

Evaluated Version (if any)

e.g. "V1", "Release 2017-03-01", "Version 2.0.27913"

Version 1 (released April 1, 2019)

Language(s) *

What language is the intervention/app in? If multiple languages are available, separate by comma (e.g. "English, French")

Version 1: Dutch. Version 2: Dutch, partially ava

URL of your Intervention Website or App

e.g. a direct link to the mobile app on app in appstore (itunes, Google Play), or URL of the website. If the intervention is a DVD or hardware, you can also link to an Amazon page.

https://www.safewomen.nl

URL of an image/screenshot (optional)

Jouw antwoord

Accessibility *

Can an enduser access the intervention presently?

- ◯ access is free and open
- ◯ access only for special usergroups, not open
- ◯ access is open to everyone, but requires payment/subscription/in-app purchases
- ◯ app/intervention no longer accessible
- ◉ Anders: access is free but registration with a username and password is mar

Primary Medical Indication/Disease/Condition *

e.g. "Stress", "Diabetes", or define the target group in brackets after the condition, e.g. "Autism (Parents of children with)", "Alzheimers (Informal Caregivers of)"

Intimate partner violence and abuse (Women v

Primary Outcomes measured in trial *

comma-separated list of primary outcomes reported in the trial

Self-efficacy

Secondary/other outcomes

Are there any other outcomes the intervention is expected to affect?

Anxiety, depression, awareness, fear of partner, perceived social support, perceived support by website, feeling helped, feasibility aspects.

Recommended "Dose" *

What do the instructions for users say on how often the app should be used?

- ( ) Approximately Daily
- ( ) Approximately Weekly
- ( ) Approximately Monthly
- ( ) Approximately Yearly
- ( ) "as needed"
- (x) Anders: no recommended dose

Approx. Percentage of Users (starters) still using the app as recommended after 3 months *

- ( ) unknown / not evaluated
- ( ) 0-10%
- ( ) 11-20%
- ( ) 21-30%
- ( ) 31-40%
- ( ) 41-50%
- ( ) 51-60%
- ( ) 61-70%
- ( ) 71%-80%
- ( ) 81-90%
- ( ) 91-100%
- (x) Anders: no recommendation for usage

Overall, was the app/intervention effective? *

- ◯ yes: all primary outcomes were significantly better in intervention group vs control
- ◯ partly: SOME primary outcomes were significantly better in intervention group vs control
- ◯ no statistically significant difference between control and intervention
- ◯ potentially harmful: control was significantly better than intervention in one or more outcomes
- ◯ inconclusive: more research is needed
- ◉ Anders: no statistically significant difference between control and interventic

Article Preparation Status/Stage *

At which stage in your article preparation are you currently (at the time you fill in this form)

- ◯ not submitted yet - in early draft status
- ◯ not submitted yet - in late draft status, just before submission
- ◯ submitted to a journal but not reviewed yet
- ◉ submitted to a journal and after receiving initial reviewer comments
- ◯ submitted to a journal and accepted, but not published yet
- ◯ published
- ◯ Anders:

Journal *

If you already know where you will submit this paper (or if it is already submitted), please provide the journal name (if it is not JMIR, provide the journal name under "other")

- ( ) not submitted yet / unclear where I will submit this
- (x) Journal of Medical Internet Research (JMIR)
- ( ) JMIR mHealth and UHealth
- ( ) JMIR Serious Games
- ( ) JMIR Mental Health
- ( ) JMIR Public Health
- ( ) JMIR Formative Research
- ( ) Other JMIR sister journal
- ( ) Anders:

Is this a full powered effectiveness trial or a pilot/feasibility trial? *

- ( ) Pilot/feasibility
- (x) Fully powered

Manuscript tracking number *

If this is a JMIR submission, please provide the manuscript tracking number under "other" (The ms tracking number can be found in the submission acknowledgement email, or when you login as author in JMIR. If the paper is already published in JMIR, then the ms tracking number is the four-digit number at the end of the DOI, to be found at the bottom of each published article in JMIR)

- ( ) no ms number (yet) / not (yet) submitted to / published in JMIR
- (x) Anders: ms#42641

TITLE AND ABSTRACT

1a) TITLE: Identification as a randomized trial in the title

1a) Does your paper address CONSORT item 1a? *

I.e does the title contain the phrase "Randomized Controlled Trial"? (if not, explain the reason under "other")

- (x) yes
- ( ) Anders:

1a-i) Identify the mode of delivery in the title

Identify the mode of delivery. Preferably use “web-based” and/or “mobile” and/or “electronic game” in the title. Avoid ambiguous terms like “online”, “virtual”, “interactive”. Use “Internet-based” only if Intervention includes non-web-based Internet components (e.g. email), use “computer-based” or “electronic” only if offline products are used. Use “virtual” only in the context of “virtual reality” (3-D worlds). Use “online” only in the context of “online support groups”. Complement or substitute product names with broader terms for the class of products (such as “mobile” or “smart phone” instead of “iphone”), especially if the application runs on different platforms.

subitem not at all important

- 1 ( )
- 2 ( )
- 3 ( )
- 4 ( )
- 5 ( )

essential

Does your paper address subitem 1a-i? *

Copy and paste relevant sections from manuscript title (include quotes in quotation marks "like this" to indicate direct quotes from your manuscript), or elaborate on this item by providing additional information not in the ms, or briefly explain why the item is not applicable/relevant for your study

Yes: "Effectiveness of the SAFE eHealth intervention for women experiencing intimate partner violence and abuse: randomized controlled trial, quantitative process evaluation, and open feasibility study."

1a-ii) Non-web-based components or important co-interventions in title

Mention non-web-based components or important co-interventions in title, if any (e.g., “with telephone support”).

subitem not at all important

1 ○

2 ○

3 ○

4 ○

5 ○

essential

Does your paper address subitem 1a-ii?

Copy and paste relevant sections from manuscript title (include quotes in quotation marks "like this" to indicate direct quotes from your manuscript), or elaborate on this item by providing additional information not in the ms, or briefly explain why the item is not applicable/relevant for your study

Not applicable as our study concerns a fully online eHealth intervention.

1a-iii) Primary condition or target group in the title

Mention primary condition or target group in the title, if any (e.g., "for children with Type I Diabetes") Example: A Web-based and Mobile Intervention with Telephone Support for Children with Type I Diabetes: Randomized Controlled Trial

subitem not at all important

1 ○

2 ○

3 ○

4 ○

5 ○

essential

Does your paper address subitem 1a-iii? *

Copy and paste relevant sections from manuscript title (include quotes in quotation marks "like this" to indicate direct quotes from your manuscript), or elaborate on this item by providing additional information not in the ms, or briefly explain why the item is not applicable/relevant for your study

Yes: "Effectiveness of the SAFE eHealth intervention for women experiencing intimate partner violence and abuse: randomized controlled trial, quantitative process evaluation, and open feasibility study."

1b) ABSTRACT: Structured summary of trial design, methods, results, and conclusions

NPT extension: Description of experimental treatment, comparator, care providers, centers, and blinding status.

1b-i) Key features/functionalities/components of the intervention and comparator in the METHODS section of the ABSTRACT

Mention key features/functionalities/components of the intervention and comparator in the abstract. If possible, also mention theories and principles used for designing the site. Keep in mind the needs of systematic reviewers and indexers by including important synonyms. (Note: Only report in the abstract what the main paper is reporting. If this information is missing from the main body of text, consider adding it)

subitem not at all important

1 ○

2 ○

3 ○

4 ○

5 ○

essential

Does your paper address subitem 1b-i? *

Copy and paste relevant sections from the manuscript abstract (include quotes in quotation marks "like this" to indicate direct quotes from your manuscript), or elaborate on this item by providing additional information not in the ms, or briefly explain why the item is not applicable/relevant for your study

Yes, a brief explanation of the content of the intervention is provided in the abstract: "They were allocated to (a) the intervention group (N=99) with access to a complete version of a help-website containing four modules on IPVA, support options, (mental) health and social support, and with interactive components such as a chat, or (b) to the limited-intervention control group (N=99)."

1b-ii) Level of human involvement in the METHODS section of the ABSTRACT

Clarify the level of human involvement in the abstract, e.g., use phrases like “fully automated” vs. “therapist/nurse/care provider/physician-assisted” (mention number and expertise of providers involved, if any). (Note: Only report in the abstract what the main paper is reporting. If this information is missing from the main body of text, consider adding it)

subitem not at all important

1 ○

2 ○

3 ○

4 ○

5 ○

essential

Does your paper address subitem 1b-ii?

Copy and paste relevant sections from the manuscript abstract (include quotes in quotation marks "like this" to indicate direct quotes from your manuscript), or elaborate on this item by providing additional information not in the ms, or briefly explain why the item is not applicable/relevant for your study

Yes, the abstract states that for the intervention arm a chat is available, showing that there is a certain degree of human contact possible: "They were allocated to (a) the intervention group (N=99) with access to a complete version of a help-website containing four modules on IPVA, support options, (mental) health and social support, and with interactive components such as a chat, or (b) to the limited-intervention control group (N=99)."

1b-iii) Open vs. closed, web-based (self-assessment) vs. face-to-face assessments in the METHODS section of the ABSTRACT

Mention how participants were recruited (online vs. offline), e.g., from an open access website or from a clinic or a closed online user group (closed usergroup trial), and clarify if this was a purely web-based trial, or there were face-to-face components (as part of the intervention or for assessment). Clearly say if outcomes were self-assessed through questionnaires (as common in web-based trials). Note: In traditional offline trials, an open trial (open-label trial) is a type of clinical trial in which both the researchers and participants know which treatment is being administered. To avoid confusion, use “blinded” or “unblinded” to indicated the level of blinding instead of “open”, as “open” in web-based trials usually refers to “open access” (i.e. participants can self-enrol). (Note: Only report in the abstract what the main paper is reporting. If this information is missing from the main body of text, consider adding it)

subitem not at all important

1 ○

2 ○

3 ○

4 ○

5 ○

essential

Does your paper address subitem 1b-iii?

Copy and paste relevant sections from the manuscript abstract (include quotes in quotation marks "like this" to indicate direct quotes from your manuscript), or elaborate on this item by providing additional information not in the ms, or briefly explain why the item is not applicable/relevant for your study

Yes: "Participants were largely recruited online and signed up through self-referral. They were allocated (blinded for the participants) to (a) the intervention group (N=99) ... or (b) to the limited-intervention control group (N=99). ... All data for this study was collected through online self-report questionnaires and automatically registered online data such as page visits and amount of logins."

1b-iv) RESULTS section in abstract must contain use data

Report number of participants enrolled/assessed in each group, the use/uptake of the intervention (e.g., attrition/adherence metrics, use over time, number of logins etc.), in addition to primary/secondary outcomes. (Note: Only report in the abstract what the main paper is reporting. If this information is missing from the main body of text, consider adding it)

subitem not at all important

1 ○

2 ○

3 ○

4 ○

5 ○

essential

Does your paper address subitem 1b-iv?

Copy and paste relevant sections from the manuscript abstract (include quotes in quotation marks "like this" to indicate direct quotes from your manuscript), or elaborate on this item by providing additional information not in the ms, or briefly explain why the item is not applicable/relevant for your study

Yes, however the division of participants in the control (N=99) and intervention (N=99) arms has been described in the Methods section of the Abstract. In the Results section: "However, we encountered high attrition for the follow-up surveys. ... The average amount of logins did not significantly differ between the study arms but participants in the intervention arm did spend significantly more time on the website. An increase of registrations during the OFS (N=170) was found: the mean amount of registrations per month was 13,2 during the RCT and 56,7 during the OFS."

1b-v) CONCLUSIONS/DISCUSSION in abstract for negative trials

Conclusions/Discussions in abstract for negative trials: Discuss the primary outcome - if the trial is negative (primary outcome not changed), and the intervention was not used, discuss whether negative results are attributable to lack of uptake and discuss reasons. (Note: Only report in the abstract what the main paper is reporting. If this information is missing from the main body of text, consider adding it)

subitem not at all important

1 ○

2 ○

3 ○

4 ○

5 ○

essential

Does your paper address subitem 1b-v?

Copy and paste relevant sections from the manuscript abstract (include quotes in quotation marks "like this" to indicate direct quotes from your manuscript), or elaborate on this item by providing additional information not in the ms, or briefly explain why the item is not applicable/relevant for your study

Yes: "Our findings did not show a significant difference in outcomes between the extensive SAFE intervention compared to the limited-intervention control group. It is, however, difficult to quantify the real contribution of the interactive components, as the control group also had access to a limited version of the intervention for ethical reasons. ... Integrated and multi-layered approaches are needed to aptly quantify the impact of online IPVA interventions for survivors."

INTRODUCTION

2a) In INTRODUCTION: Scientific background and explanation of rationale

2a-i) Problem and the type of system/solution

Describe the problem and the type of system/solution that is object of the study: intended as stand-alone intervention vs. incorporated in broader health care program? Intended for a particular patient population? Goals of the intervention, e.g., being more cost-effective to other interventions, replace or complement other solutions? (Note: Details about the intervention are provided in “Methods” under 5)

subitem not at all important

1 ○

2 ○

3 ○

4 ○

5 ○

essential

Does your paper address subitem 2a-i? *

Copy and paste relevant sections from the manuscript (include quotes in quotation marks "like this" to indicate direct quotes from your manuscript), or elaborate on this item by providing additional information not in the ms, or briefly explain why the item is not applicable/relevant for your study

Yes, for example: "Despite their frequent occurrence, IPVA and DVA are still taboo subjects and survivors often struggle to disclose the violence or seek help due to shame, fear, stigma, feeling guilty, not recognizing IPVA, financial dependency, residency permit dependency, and / or children being involved [7-9]. Furthermore, lockdowns and mitigation measures during the COVID-19 pandemic [10], that occurred simultaneously with a part of this study, not only exacerbated IPVA but also heightened the barriers for disclosing and help seeking [11-14]. ... Indeed, online tools and interventions can increase accessibility of support and help options. eHealth is still relatively new in the field of IPVA research, but several studies showed the feasibility and effectiveness that online interventions have in supporting IPVA survivors."

2a-ii) Scientific background, rationale: What is known about the (type of) system

Scientific background, rationale: What is known about the (type of) system that is the object of the study (be sure to discuss the use of similar systems for other conditions/diagnoses, if appropiate), motivation for the study, i.e. what are the reasons for and what is the context for this specific study, from which stakeholder viewpoint is the study performed, potential impact of findings [2]. Briefly justify the choice of the comparator.

subitem not at all important

1 ◯

2 ◯

3 ◯

4 ◯

5 ◯

essential

Does your paper address subitem 2a-ii? *

Copy and paste relevant sections from the manuscript (include quotes in quotation marks "like this" to indicate direct quotes from your manuscript), or elaborate on this item by providing additional information not in the ms, or briefly explain why the item is not applicable/relevant for your study

Yes, for example: "eHealth is still relatively new in the field of IPVA research, but several studies showed the feasibility and effectiveness that online interventions have in supporting IPVA survivors. For example, in improving mental health and decreasing exposure to IPVA and in increasing awareness, safety behaviors, and feeling supported [21-27]. ... All the aforementioned outcomes for eHealth interventions in the IPVA context originate from Australia, Canada, the United States, and New-Zealand. In Europe, our team in the Netherlands developed the first eHealth intervention for female IPVA survivors that is scientifically evaluated through a randomized controlled trial (RCT), a process evaluation (PE), and an open feasibility study (OFS). The Dutch online intervention SAFE (www.safewomen.nl) was inspired by the Australian I-DECIDE intervention [29] and the Dutch Feel the Vibe intervention [30], and based on scientific knowledge and the insights from Dutch IPVA survivors and professionals [31]."

2b) In INTRODUCTION: Specific objectives or hypotheses

Does your paper address CONSORT subitem 2b? *

Copy and paste relevant sections from the manuscript (include quotes in quotation marks "like this" to indicate direct quotes from your manuscript), or elaborate on this item by providing additional information not in the ms, or briefly explain why the item is not applicable/relevant for your study

Yes, research questions: "The primary research question is: - Is SAFE more effective in increasing self-efficacy in women exposed to IPVA than a minimal intervention? Secondary research questions are: - Is SAFE an effective intervention to increase awareness and perceived support and to lower symptoms of mental health problems in women exposed to IPVA? - Is SAFE a feasible tool in the real-world setting for providing information and support to women exposed to IPVA?"

METHODS

3a) Description of trial design (such as parallel, factorial) including allocation ratio

Does your paper address CONSORT subitem 3a? *

Copy and paste relevant sections from the manuscript (include quotes in quotation marks "like this" to indicate direct quotes from your manuscript), or elaborate on this item by providing additional information not in the ms, or briefly explain why the item is not applicable/relevant for your study

Yes: "The RCT is a parallel-group design with two arms and stratified (block size of four) automated randomization in two age groups (18 – 30 years old; 31 – 50 years old). The eHealth developer and a statistician generated this random allocation sequence. The randomization was single-blinded for the participants."

3b) Important changes to methods after trial commencement (such as eligibility criteria), with reasons

Does your paper address CONSORT subitem 3b? *

Copy and paste relevant sections from the manuscript (include quotes in quotation marks "like this" to indicate direct quotes from your manuscript), or elaborate on this item by providing additional information not in the ms, or briefly explain why the item is not applicable/relevant for your study

Yes, see Methods - Ethics Approval: "All research components, including two amendments, with regard to clarifying the inclusion criteria for scores on fear of partner and introducing the open feasibility study, were approved by the Medical Ethics Committee from Arnhem and Nijmegen (NL68268.091.18; dossier 2018-5009) ..."

3b-i) Bug fixes, Downtimes, Content Changes

Bug fixes, Downtimes, Content Changes: ehealth systems are often dynamic systems. A description of changes to methods therefore also includes important changes made on the intervention or comparator during the trial (e.g., major bug fixes or changes in the functionality or content) (5-iii) and other “unexpected events” that may have influenced study design such as staff changes, system failures/downtimes, etc. [2].

subitem not at all important

1 ○

2 ○

3 ○

4 ○

5 ○

essential

Does your paper address subitem 3b-i?

Copy and paste relevant sections from the manuscript (include quotes in quotation marks "like this" to indicate direct quotes from your manuscript), or elaborate on this item by providing additional information not in the ms, or briefly explain why the item is not applicable/relevant for your study

No important changes were made to the intervention during the RCT and downtimes did not occur.

4a) Eligibility criteria for participants

Does your paper address CONSORT subitem 4a? *

Copy and paste relevant sections from the manuscript (include quotes in quotation marks "like this" to indicate direct quotes from your manuscript), or elaborate on this item by providing additional information not in the ms, or briefly explain why the item is not applicable/relevant for your study

Yes: "Women in the RCT were between 18 and 50 years old that had a sufficient comprehension of Dutch and that experienced IPVA no longer than one year ago or still experienced significant fear of their partner."

4a-i) Computer / Internet literacy

Computer / Internet literacy is often an implicit “de facto” eligibility criterion - this should be explicitly clarified.

subitem not at all important

1 ○

2 ○

3 ○

4 ○

5 ○

essential

Does your paper address subitem 4a-i?

Copy and paste relevant sections from the manuscript (include quotes in quotation marks "like this" to indicate direct quotes from your manuscript), or elaborate on this item by providing additional information not in the ms, or briefly explain why the item is not applicable/relevant for your study

This is explicitly stated in the earlier published protocol (https://bmcpublichealth.biomedcentral.com/articles/10.1186/s12889-020-08743-0) For this article: "The participants for the RCT were largely recruited online ... All participants digitally received an information letter and signed an informed consent form through checking a box. ... Also, while many people in the Netherlands have access to the internet (97%) and are digitally literate (50% has 'above basic overall digital skills') [74,75], women who did not have access to the internet or who do not know how to use it were excluded."

4a-ii) Open vs. closed, web-based vs. face-to-face assessments:

Open vs. closed, web-based vs. face-to-face assessments: Mention how participants were recruited (online vs. offline), e.g., from an open access website or from a clinic, and clarify if this was a purely web-based trial, or there were face-to-face components (as part of the intervention or for assessment), i.e., to what degree got the study team to know the participant. In online-only trials, clarify if participants were quasi-anonymous and whether having multiple identities was possible or whether technical or logistical measures (e.g., cookies, email confirmation, phone calls) were used to detect/prevent these.

subitem not at all important

1 ○

2 ○

3 ○

4 ○

5 ○

essential

Does your paper address subitem 4a-ii? *

Copy and paste relevant sections from the manuscript (include quotes in quotation marks "like this" to indicate direct quotes from your manuscript), or elaborate on this item by providing additional information not in the ms, or briefly explain why the item is not applicable/relevant for your study

Yes: "Participants were largely recruited online and signed up through self-referral. They were allocated (blinded for the participants) to (a) the intervention group (N=99) ... or (b) to the limited-intervention control group (N=99)." Participants needed to use their e-mail address to register and filled out a survey on demographics: sex, gender, age, sexual orientation, country of birth, cultural identification, religious identification, educational level, income, employment, children (yes/no). They never gave their name and thus could remain anonymous. More information is present in the earlier published protocol (https://bmcpublichealth.biomedcentral.com/articles/10.1186/s12889-020-08743-0): community managers monitored the registrations and communication via chat and forum to intercept suspicious or clear signs of people pretending to be someone else and posing a possible threat, no such incidents happened during the RCT.

4a-iii) Information giving during recruitment

Information given during recruitment. Specify how participants were briefed for recruitment and in the informed consent procedures (e.g., publish the informed consent documentation as appendix, see also item X26), as this information may have an effect on user self-selection, user expectation and may also bias results.

subitem not at all important

1 ○

2 ○

3 ○

4 ○

5 ○

essential

Does your paper address subitem 4a-iii?

Copy and paste relevant sections from the manuscript (include quotes in quotation marks "like this" to indicate direct quotes from your manuscript), or elaborate on this item by providing additional information not in the ms, or briefly explain why the item is not applicable/relevant for your study

Yes: "All participants digitally received an information letter and signed an informed consent form through checking a box. Subsequently, we enforced a mandatory 24-hour waiting period to ensure participants had sufficient time to contemplate their decision to participate. Participants then provided digital consent again, filled out the M0 (baseline) questionnaires, and were randomized in the control or intervention arm."

4b) Settings and locations where the data were collected

Does your paper address CONSORT subitem 4b? *

Copy and paste relevant sections from the manuscript (include quotes in quotation marks "like this" to indicate direct quotes from your manuscript), or elaborate on this item by providing additional information not in the ms, or briefly explain why the item is not applicable/relevant for your study

Yes: "All data for this study was collected through online self-report questionnaires and automatically registered online data such as page visits and amount of logins."

4b-i) Report if outcomes were (self-)assessed through online questionnaires

Clearly report if outcomes were (self-)assessed through online questionnaires (as common in web-based trials) or otherwise.

subitem not at all important

1 ○

2 ○

3 ○

4 ○

5 ○

essential

Does your paper address subitem 4b-i? *

Copy and paste relevant sections from the manuscript (include quotes in quotation marks "like this" to indicate direct quotes from your manuscript), or elaborate on this item by providing additional information not in the ms, or briefly explain why the item is not applicable/relevant for your study

Yes: "The outcomes were assessed with online self-report questionnaires at the registration process (M0), at three (M3), six (M6), and 12 months (M12) (Multimedia Appendix 1; source: [32])."

4b-ii) Report how institutional affiliations are displayed

Report how institutional affiliations are displayed to potential participants [on ehealth media], as affiliations with prestigious hospitals or universities may affect volunteer rates, use, and reactions with regards to an intervention.(Not a required item – describe only if this may bias results)

subitem not at all important

1 ○

2 ○

3 ○

4 ○

5 ○

essential

Does your paper address subitem 4b-ii?

Copy and paste relevant sections from the manuscript (include quotes in quotation marks "like this" to indicate direct quotes from your manuscript), or elaborate on this item by providing additional information not in the ms, or briefly explain why the item is not applicable/relevant for your study

Yes: "Through the information letter and a statement on the intervention website, participants were aware that this study was conducted by researchers from the Radboudumc."

5) The interventions for each group with sufficient details to allow replication, including how and when they were actually administered

5-i) Mention names, credential, affiliations of the developers, sponsors, and owners

Mention names, credential, affiliations of the developers, sponsors, and owners [6] (if authors/evaluators are owners or developer of the software, this needs to be declared in a “Conflict of interest” section or mentioned elsewhere in the manuscript).

subitem not at all important

1 ○

2 ○

3 ○

4 ○

5 ○

essential

Does your paper address subitem 5-i?

Copy and paste relevant sections from the manuscript (include quotes in quotation marks "like this" to indicate direct quotes from your manuscript), or elaborate on this item by providing additional information not in the ms, or briefly explain why the item is not applicable/relevant for your study

Yes: "Lastly, we would like to acknowledge the eHealth developer, Ippo, and our funder, ZonMw. This study is government funded by the Gender and Health program of ZonMw (grant number 849200002)."

5-ii) Describe the history/development process

Describe the history/development process of the application and previous formative evaluations (e.g., focus groups, usability testing), as these will have an impact on adoption/use rates and help with interpreting results.

subitem not at all important

1 ○

2 ○

3 ○

4 ○

5 ○

essential

Does your paper address subitem 5-ii?

Copy and paste relevant sections from the manuscript (include quotes in quotation marks "like this" to indicate direct quotes from your manuscript), or elaborate on this item by providing additional information not in the ms, or briefly explain why the item is not applicable/relevant for your study

The development process is described in another study: https://journals.sagepub.com/doi/10.1177/08862605211036108. For this article: "The Dutch online intervention SAFE (www.safewomen.nl) was inspired by the Australian I-DECIDE intervention [29] and the Dutch Feel the Vibe intervention [30], and based on scientific knowledge and the insights from Dutch IPVA survivors and professionals [31]. The development process of the intervention and the study protocol for the RCT, PE, and OFS are available elsewhere [31-32]."

5-iii) Revisions and updating

Revisions and updating. Clearly mention the date and/or version number of the application/intervention (and comparator, if applicable) evaluated, or describe whether the intervention underwent major changes during the evaluation process, or whether the development and/or content was “frozen” during the trial. Describe dynamic components such as news feeds or changing content which may have an impact on the replicability of the intervention (for unexpected events see item 3b).

subitem not at all important

1 ○

2 ○

3 ○

4 ○

5 ○

essential

Does your paper address subitem 5-iii?

Copy and paste relevant sections from the manuscript (include quotes in quotation marks "like this" to indicate direct quotes from your manuscript), or elaborate on this item by providing additional information not in the ms, or briefly explain why the item is not applicable/relevant for your study

Yes: "The intervention was frozen during the RCT, meaning no major changes to the intervention were made during the trial."

5-iv) Quality assurance methods

Provide information on quality assurance methods to ensure accuracy and quality of information provided [1], if applicable.

subitem not at all important

1 ○

2 ○

3 ○

4 ○

5 ○

essential

Does your paper address subitem 5-iv?

Copy and paste relevant sections from the manuscript (include quotes in quotation marks "like this" to indicate direct quotes from your manuscript), or elaborate on this item by providing additional information not in the ms, or briefly explain why the item is not applicable/relevant for your study

We used validated questionnaires (see Multimedia Appendix 1: Primary and secondary outcome measures and measurement timepoints.).

5-v) Ensure replicability by publishing the source code, and/or providing screenshots/screen-capture video, and/or providing flowcharts of the algorithms used

Ensure replicability by publishing the source code, and/or providing screenshots/screen-capture video, and/or providing flowcharts of the algorithms used. Replicability (i.e., other researchers should in principle be able to replicate the study) is a hallmark of scientific reporting.

subitem not at all important

1

2

3

4

5

essential

Does your paper address subitem 5-v?

Copy and paste relevant sections from the manuscript (include quotes in quotation marks "like this" to indicate direct quotes from your manuscript), or elaborate on this item by providing additional information not in the ms, or briefly explain why the item is not applicable/relevant for your study

This data is not available.

5-vi) Digital preservation

Digital preservation: Provide the URL of the application, but as the intervention is likely to change or disappear over the course of the years; also make sure the intervention is archived (Internet Archive, webcitation.org, and/or publishing the source code or screenshots/videos alongside the article). As pages behind login screens cannot be archived, consider creating demo pages which are accessible without login.

subitem not at all important

1 ○

2 ○

3 ○

4 ○

5 ○

essential

Does your paper address subitem 5-vi?

Copy and paste relevant sections from the manuscript (include quotes in quotation marks "like this" to indicate direct quotes from your manuscript), or elaborate on this item by providing additional information not in the ms, or briefly explain why the item is not applicable/relevant for your study

https://www.safewomen.nl; demo pages are not available.

5-vii) Access

Access: Describe how participants accessed the application, in what setting/context, if they had to pay (or were paid) or not, whether they had to be a member of specific group. If known, describe how participants obtained “access to the platform and Internet” [1]. To ensure access for editors/reviewers/readers, consider to provide a “backdoor” login account or demo mode for reviewers/readers to explore the application (also important for archiving purposes, see vi).

subitem not at all important

1 ○

2 ○

3 ○

4 ○

5 ○

essential

Does your paper address subitem 5-vii? *

Copy and paste relevant sections from the manuscript (include quotes in quotation marks "like this" to indicate direct quotes from your manuscript), or elaborate on this item by providing additional information not in the ms, or briefly explain why the item is not applicable/relevant for your study

Yes: "The participants for the RCT were largely recruited online, between April 1st 2019 and October 1st 2020, and signed up through self-referral or via a DVA, social, or (mental) healthcare professional. Women in the RCT were between 18 and 50 years old that had a sufficient comprehension of Dutch and that experienced IPVA no longer than one year ago or still experienced significant fear of their partner."

5-viii) Mode of delivery, features/functionalities/components of the intervention and comparator, and the theoretical framework

Describe mode of delivery, features/functionalities/components of the intervention and comparator, and the theoretical framework [6] used to design them (instructional strategy [1], behaviour change techniques, persuasive features, etc., see e.g., [7, 8] for terminology). This includes an in-depth description of the content (including where it is coming from and who developed it) [1],” whether [and how] it is tailored to individual circumstances and allows users to track their progress and receive feedback” [6]. This also includes a description of communication delivery channels and – if computer-mediated communication is a component – whether communication was synchronous or asynchronous [6]. It also includes information on presentation strategies [1], including page design principles, average amount of text on pages, presence of hyperlinks to other resources, etc. [1].

subitem not at all important

1 ○

2 ○

3 ○

4 ○

5 ○

essential

Does your paper address subitem 5-viii? *

Copy and paste relevant sections from the manuscript (include quotes in quotation marks "like this" to indicate direct quotes from your manuscript), or elaborate on this item by providing additional information not in the ms, or briefly explain why the item is not applicable/relevant for your study

Yes, for example: "The RCT intervention group received the complete intervention: access to a website with support for IPVA survivors with interactive components. The control group received the minimal intervention with only the most essential static information (Table 1)."
Table 1: SAFE modules and functionalities during RCT and OFS. The earlier published protocol (https://bmcpublichealth.biomedcentral.com/articles/10.1186/s12889-020-08743-0) provides additional information on the intervention.

5-ix) Describe use parameters

Describe use parameters (e.g., intended “doses” and optimal timing for use). Clarify what instructions or recommendations were given to the user, e.g., regarding timing, frequency, heaviness of use, if any, or was the intervention used ad libitum.

subitem not at all important

1 ○

2 ○

3 ○

4 ○

5 ○

essential

Does your paper address subitem 5-ix?

Copy and paste relevant sections from the manuscript (include quotes in quotation marks "like this" to indicate direct quotes from your manuscript), or elaborate on this item by providing additional information not in the ms, or briefly explain why the item is not applicable/relevant for your study

Not applicable, no recommendations for usage were given.

5-x) Clarify the level of human involvement

Clarify the level of human involvement (care providers or health professionals, also technical assistance) in the e-intervention or as co-intervention (detail number and expertise of professionals involved, if any, as well as “type of assistance offered, the timing and frequency of the support, how it is initiated, and the medium by which the assistance is delivered”. It may be necessary to distinguish between the level of human involvement required for the trial, and the level of human involvement required for a routine application outside of a RCT setting (discuss under item 21 – generalizability).

subitem not at all important

1 ○

2 ○

3 ○

4 ○

5 ○

essential

Does your paper address subitem 5-x?

Copy and paste relevant sections from the manuscript (include quotes in quotation marks "like this" to indicate direct quotes from your manuscript), or elaborate on this item by providing additional information not in the ms, or briefly explain why the item is not applicable/relevant for your study

Yes, see Table 1 SAFE modules and functionalities during RCT and OFS: "Contact: - Links to contact options with fellow survivors. - Option to contact the community managers. - Chat, forum, diary." Furthermore: "They were allocated (blinded for the participants) to (a) the intervention group (N=99) with access to a complete version of a help-website withcontaining four modules on IPVA, support options, (mental) health and social support, and with interactive components such as a chat, ..." And supplementary material: Multimedia Appendix 4. Feasibility data from Matomo and SAFE’s social media accounts and messages.

5-xi) Report any prompts/reminders used

Report any prompts/reminders used: Clarify if there were prompts (letters, emails, phone calls, SMS) to use the application, what triggered them, frequency etc. It may be necessary to distinguish between the level of prompts/reminders required for the trial, and the level of prompts/reminders for a routine application outside of a RCT setting (discuss under item 21 – generalizability).

subitem not at all important

1 ○

2 ○

3 ○

4 ○

5 ○

essential

Does your paper address subitem 5-xi? *

Copy and paste relevant sections from the manuscript (include quotes in quotation marks "like this" to indicate direct quotes from your manuscript), or elaborate on this item by providing additional information not in the ms, or briefly explain why the item is not applicable/relevant for your study

Yes, see Multimedia Appendix 1. Primary and secondary outcome measures and measurement timepoints: "Note: participants received a maximum of two e-mail reminders if they did not complete a questionnaire timepoint."

5-xii) Describe any co-interventions (incl. training/support)

Describe any co-interventions (incl. training/support): Clearly state any interventions that are provided in addition to the targeted eHealth intervention, as ehealth intervention may not be designed as stand-alone intervention. This includes training sessions and support [1]. It may be necessary to distinguish between the level of training required for the trial, and the level of training for a routine application outside of a RCT setting (discuss under item 21 – generalizability.

subitem not at all important

1 ○

2 ○

3 ○

4 ○

5 ○

essential

Does your paper address subitem 5-xii? *

Copy and paste relevant sections from the manuscript (include quotes in quotation marks "like this" to indicate direct quotes from your manuscript), or elaborate on this item by providing additional information not in the ms, or briefly explain why the item is not applicable/relevant for your study

Not applicable, no co-interventions were provided.

6a) Completely defined pre-specified primary and secondary outcome measures, including how and when they were assessed

Does your paper address CONSORT subitem 6a? *

Copy and paste relevant sections from the manuscript (include quotes in quotation marks "like this" to indicate direct quotes from your manuscript), or elaborate on this item by providing additional information not in the ms, or briefly explain why the item is not applicable/relevant for your study

Yes: Multimedia Appendix 1. Primary and secondary outcome measures and measurement timepoints.

6a-i) Online questionnaires: describe if they were validated for online use and apply CHERRIES items to describe how the questionnaires were designed/deployed

If outcomes were obtained through online questionnaires, describe if they were validated for online use and apply CHERRIES items to describe how the questionnaires were designed/deployed [9].

subitem not at all important

1 ○

2 ○

3 ○

4 ○

5 ○

essential

Does your paper address subitem 6a-i?

Copy and paste relevant sections from manuscript text

Yes, we provide information on CHERRIES aspect such as target population, ethical approval, informed consent, recruitment process, access to the questionnaires, response rates etc. See for example 'Table 3. ANCOVA and GEE for self-efficacy and the secondary outcomes' that shows the response rates for the primary outcome (self-efficacy) at M0 (N=198) and M6 (N=42).

6a-ii) Describe whether and how “use” (including intensity of use/dosage) was defined/measured/monitored

Describe whether and how “use” (including intensity of use/dosage) was defined/measured/monitored (logins, logfile analysis, etc.). Use/adoption metrics are important process outcomes that should be reported in any ehealth trial.

subitem not at all important

1 ○

2 ○

3 ○

4 ○

5 ○

essential

Does your paper address subitem 6a-ii?

Copy and paste relevant sections from manuscript text

Yes, we registered amount of logins, time spent on the website, visited pages: "The RCT user data showed that 22 participants in the intervention group and 26 participants in the control group never logged in (Multimedia Appendix 3). The intervention group spent significantly more time on the intervention than the control group (p<.01) but we found no significant difference between the study arms for the average amount of logins (p=.078). Women in the RCT intervention group and OFS mostly visited the interactive contact options, such as the forum. The RCT control group mostly used the pages on help options (Multimedia Appendix 3)." (Multimedia Appendix 3. User data from the RCT study arms and OFS group.)

6a-iii) Describe whether, how, and when qualitative feedback from participants was obtained

Describe whether, how, and when qualitative feedback from participants was obtained (e.g., through emails, feedback forms, interviews, focus groups).

subitem not at all important

1 ○

2 ○

3 ○

4 ○

5 ○

essential

Does your paper address subitem 6a-iii?

Copy and paste relevant sections from manuscript text

Yes, it refers to the other article on the qualitative evaluation of this intervention, which is currently in the review process. "Our corresponding qualitative evaluation [36] shows that while the intervention did not always explicitly improve self-efficacy or mental health problems or show significant statistical differences, women did find it helpful in terms of awareness, support, and acknowledgement, and they were satisfied with the provided information and help options."

6b) Any changes to trial outcomes after the trial commenced, with reasons

Does your paper address CONSORT subitem 6b? *

Copy and paste relevant sections from the manuscript (include quotes in quotation marks "like this" to indicate direct quotes from your manuscript), or elaborate on this item by providing additional information not in the ms, or briefly explain why the item is not applicable/relevant for your study

Not applicable, no changes to trial outcomes were made after the trial commenced.

7a) How sample size was determined

NPT: When applicable, details of whether and how the clustering by care provides or centers was addressed

7a-i) Describe whether and how expected attrition was taken into account when calculating the sample size

Describe whether and how expected attrition was taken into account when calculating the sample size.

subitem not at all important

1 ○

2 ○

3 ○

4 ○

5 ○

essential

Does your paper address subitem 7a-i?

Copy and paste relevant sections from manuscript title (include quotes in quotation marks "like this" to indicate direct quotes from your manuscript), or elaborate on this item by providing additional information not in the ms, or briefly explain why the item is not applicable/relevant for your study

Yes: "The sample size was calculated based on the primary outcome measure, self-efficacy at six months, as described in the study protocol [32]."

7b) When applicable, explanation of any interim analyses and stopping guidelines

Does your paper address CONSORT subitem 7b? *

Copy and paste relevant sections from the manuscript (include quotes in quotation marks "like this" to indicate direct quotes from your manuscript), or elaborate on this item by providing additional information not in the ms, or briefly explain why the item is not applicable/relevant for your study

Interim analyses and stopping guidelines were not applicable to this study.

8a) Method used to generate the random allocation sequence

NPT: When applicable, how care providers were allocated to each trial group

Does your paper address CONSORT subitem 8a? *

Copy and paste relevant sections from the manuscript (include quotes in quotation marks "like this" to indicate direct quotes from your manuscript), or elaborate on this item by providing additional information not in the ms, or briefly explain why the item is not applicable/relevant for your study

Yes: "The RCT is a parallel-group design with two arms and stratified (block size of four) automated randomization in two age groups (18 – 30 years old; 31 – 50 years old). The eHealth developer and a statistician generated this random allocation sequence. The randomization was single-blinded for the participants. The researchers could track which participant was part of the control or intervention group but they did not have any influence on the randomization process."

8b) Type of randomisation; details of any restriction (such as blocking and block size)

Does your paper address CONSORT subitem 8b? *

Copy and paste relevant sections from the manuscript (include quotes in quotation marks "like this" to indicate direct quotes from your manuscript), or elaborate on this item by providing additional information not in the ms, or briefly explain why the item is not applicable/relevant for your study

Yes: "The RCT is a parallel-group design with two arms and stratified (block size of four) automated randomization in two age groups (18 – 30 years old; 31 – 50 years old). The eHealth developer and a statistician generated this random allocation sequence. The randomization was single-blinded for the participants. The researchers could track which participant was part of the control or intervention group but they did not have any influence on the randomization process."

9) Mechanism used to implement the random allocation sequence (such as sequentially numbered containers), describing any steps taken to conceal the sequence until interventions were assigned

Does your paper address CONSORT subitem 9? *

Copy and paste relevant sections from the manuscript (include quotes in quotation marks "like this" to indicate direct quotes from your manuscript), or elaborate on this item by providing additional information not in the ms, or briefly explain why the item is not applicable/relevant for your study

Yes: "The RCT is a parallel-group design with two arms and stratified (block size of four) automated randomization in two age groups (18 – 30 years old; 31 – 50 years old). The eHealth developer and a statistician generated this random allocation sequence. The randomization was single-blinded for the participants. The researchers could track which participant was part of the control or intervention group but they did not have any influence on the randomization process."

10) Who generated the random allocation sequence, who enrolled participants, and who assigned participants to interventions

Does your paper address CONSORT subitem 10? *

Copy and paste relevant sections from the manuscript (include quotes in quotation marks "like this" to indicate direct quotes from your manuscript), or elaborate on this item by providing additional information not in the ms, or briefly explain why the item is not applicable/relevant for your study

Yes: "The RCT is a parallel-group design with two arms and stratified (block size of four) automated randomization in two age groups (18 – 30 years old; 31 – 50 years old). The eHealth developer and a statistician generated this random allocation sequence. The randomization was single-blinded for the participants. The researchers could track which participant was part of the control or intervention group but they did not have any influence on the randomization process."

11a) If done, who was blinded after assignment to interventions (for example, participants, care providers, those assessing outcomes) and how

NPT: Whether or not administering co-interventions were blinded to group assignment

11a-i) Specify who was blinded, and who wasn't

Specify who was blinded, and who wasn't. Usually, in web-based trials it is not possible to blind the participants [1, 3] (this should be clearly acknowledged), but it may be possible to blind outcome assessors, those doing data analysis or those administering co-interventions (if any).

subitem not at all important

1 ○

2 ○

3 ○

4 ○

5 ○

essential

Does your paper address subitem 11a-i? *

Copy and paste relevant sections from the manuscript (include quotes in quotation marks "like this" to indicate direct quotes from your manuscript), or elaborate on this item by providing additional information not in the ms, or briefly explain why the item is not applicable/relevant for your study

Yes: " The randomization was single-blinded for the participants. The researchers could track which participant was part of the control or intervention group but they did not have any influence on the randomization process."

11a-ii) Discuss e.g., whether participants knew which intervention was the “intervention of interest” and which one was the “comparator”

Informed consent procedures (4a-ii) can create biases and certain expectations - discuss e.g., whether participants knew which intervention was the “intervention of interest” and which one was the “comparator”.

subitem not at all important

1 ○

2 ○

3 ○

4 ○

5 ○

essential

Does your paper address subitem 11a-ii?

Copy and paste relevant sections from the manuscript (include quotes in quotation marks "like this" to indicate direct quotes from your manuscript), or elaborate on this item by providing additional information not in the ms, or briefly explain why the item is not applicable/relevant for your study

Yes: "The randomization was single-blinded for the participants." The participants were not aware of the arm they were assigned to or which intervention was the 'intervention of interest'.

11b) If relevant, description of the similarity of interventions
(this item is usually not relevant for ehealth trials as it refers to similarity of a placebo or sham intervention to a active medication/intervention)

Does your paper address CONSORT subitem 11b? *

Copy and paste relevant sections from the manuscript (include quotes in quotation marks "like this" to indicate direct quotes from your manuscript), or elaborate on this item by providing additional information not in the ms, or briefly explain why the item is not applicable/relevant for your study

Yes, Table 1. SAFE modules and functionalities during RCT and OFS describes the similarities and differences between the intervention that the control arm received and the intervention that the intervention arm received.

12a) Statistical methods used to compare groups for primary and secondary outcomes
NPT: When applicable, details of whether and how the clustering by care providers or centers was addressed

Does your paper address CONSORT subitem 12a? *

Copy and paste relevant sections from the manuscript (include quotes in quotation marks "like this" to indicate direct quotes from your manuscript), or elaborate on this item by providing additional information not in the ms, or briefly explain why the item is not applicable/relevant for your study

Yes: "The data was analyzed with descriptive statistics (based on intention to treat), ANCOVAs, and Generalized Estimated Equations (GEEs) for the primary and secondary outcomes, controlling for baseline scores and using SPSS, version 25 [35] (Table 3). For self-efficacy, a complete case analysis (CCA) was conducted as well for M0, M3 and M6."

12a-i) Imputation techniques to deal with attrition / missing values

Imputation techniques to deal with attrition / missing values: Not all participants will use the intervention/comparator as intended and attrition is typically high in ehealth trials. Specify how participants who did not use the application or dropped out from the trial were treated in the statistical analysis (a complete case analysis is strongly discouraged, and simple imputation techniques such as LOCF may also be problematic [4]).

subitem not at all important

1 ○

2 ○

3 ○

4 ○

5 ○

essential

Does your paper address subitem 12a-i? *

Copy and paste relevant sections from the manuscript (include quotes in quotation marks "like this" to indicate direct quotes from your manuscript), or elaborate on this item by providing additional information not in the ms, or briefly explain why the item is not applicable/relevant for your study

Yes: "Due to a high dropout rate on follow-up questionnaires (M0 = 198; M6 = 42), we performed an extensive check for selective attrition bias at the M6 General Self-Efficacy (GSE) survey, using various variables on demographics, study arm, IPVA experiences, and the primary and secondary measures at baseline (Multimedia Appendix 5)."

12b) Methods for additional analyses, such as subgroup analyses and adjusted analyses

Does your paper address CONSORT subitem 12b? *

Copy and paste relevant sections from the manuscript (include quotes in quotation marks "like this" to indicate direct quotes from your manuscript), or elaborate on this item by providing additional information not in the ms, or briefly explain why the item is not applicable/relevant for your study

Yes: "The data was analyzed with descriptive statistics (based on intention to treat), ANCOVAs, and Generalized Estimated Equations (GEEs) for the primary and secondary outcomes, controlling for baseline scores and using SPSS, version 25 [35] (Table 3). For self-efficacy, a complete case analysis (CCA) was conducted as well for M0, M3 and M6."

X26) REB/IRB Approval and Ethical Considerations [recommended as subheading under "Methods"] (not a CONSORT item)

X26-i) Comment on ethics committee approval

subitem not at all important

1 ○

2 ○

3 ○

4 ○

5 ○

essential

Does your paper address subitem X26-i?

Copy and paste relevant sections from the manuscript (include quotes in quotation marks "like this" to indicate direct quotes from your manuscript), or elaborate on this item by providing additional information not in the ms, or briefly explain why the item is not applicable/relevant for your study

Yes: "All research components, including two amendments, with regard to clarifying the inclusion criteria for scores on fear of partner and introducing the open feasibility study, were approved by the Medical Ethics Committee from Arnhem and Nijmegen (NL68268.091.18; dossier 2018-5009) and the RCT was registered at the Netherlands Trial Register NL7108 (NTR7313)."

x26-ii) Outline informed consent procedures

Outline informed consent procedures e.g., if consent was obtained offline or online (how? Checkbox, etc.?), and what information was provided (see 4a-ii). See [6] for some items to be included in informed consent documents.

subitem not at all important

1 ○

2 ○

3 ○

4 ○

5 ○

essential

Does your paper address subitem X26-ii?

Copy and paste relevant sections from the manuscript (include quotes in quotation marks "like this" to indicate direct quotes from your manuscript), or elaborate on this item by providing additional information not in the ms, or briefly explain why the item is not applicable/relevant for your study

Yes: "The participants for the RCT were largely recruited online, between April 1st 2019 and October 1st 2020, and signed up through self-referral or via a DVA, social, or (mental) healthcare professional. Women in the RCT were between 18 and 50 years old that had a sufficient comprehension of Dutch and that experienced IPVA no longer than one year ago or still experienced significant fear of their partner. All participants digitally received an information letter and signed an informed consent form through checking a box. Through the information letter and a statement on the intervention website, participants were aware that this study was conducted by researchers from the Radboudumc. Subsequently, we enforced a mandatory 24-hour waiting period to ensure participants had sufficient time to contemplate their decision to participate. Participants then provided digital consent again, filled out the M0 (baseline) questionnaires, and were randomized in the control or intervention arm."

X26-iii) Safety and security procedures

Safety and security procedures, incl. privacy considerations, and any steps taken to reduce the likelihood or detection of harm (e.g., education and training, availability of a hotline)

subitem not at all important

- 1 ○
- 2 ○
- 3 ○
- 4 ○
- 5 ○

essential

Does your paper address subitem X26-iii?

Copy and paste relevant sections from the manuscript (include quotes in quotation marks "like this" to indicate direct quotes from your manuscript), or elaborate on this item by providing additional information not in the ms, or briefly explain why the item is not applicable/relevant for your study

No but this was already elaborately discussed in the earlier published protocol: https://bmcpublichealth.biomedcentral.com/articles/10.1186/s12889-020-08743-0

RESULTS

13a) For each group, the numbers of participants who were randomly assigned, received intended treatment, and were analysed for the primary outcome

NPT: The number of care providers or centers performing the intervention in each group and the number of patients treated by each care provider in each center

Does your paper address CONSORT subitem 13a? *

Copy and paste relevant sections from the manuscript (include quotes in quotation marks "like this" to indicate direct quotes from your manuscript), or elaborate on this item by providing additional information not in the ms, or briefly explain why the item is not applicable/relevant for your study

Yes: "During the RCT inclusion period, 239 out of 502 women completed the registration they started and 198 women were included in the RCT. Four participants actively dropped out during their follow-up trajectory (control N=1; intervention N=3), however attrition on follow-up questionnaires was much higher (Figure 1)." Figure 1: Registration flowchart during the RCT period (2019 – 2021) shows N=99 in the control arm and N=99 in the intervention arm.

13b) For each group, losses and exclusions after randomisation, together with reasons

Does your paper address CONSORT subitem 13b? (NOTE: Preferably, this is shown in a CONSORT flow diagram) *

Copy and paste relevant sections from the manuscript (include quotes in quotation marks "like this" to indicate direct quotes from your manuscript), or elaborate on this item by providing additional information not in the ms, or briefly explain why the item is not applicable/relevant for your study

Yes: "During the RCT inclusion period, 239 out of 502 women completed the registration they started and 198 women were included in the RCT. Four participants actively dropped out during their follow-up trajectory (control N=1; intervention N=3), however attrition on follow-up questionnaires was much higher (Figure 1)." And see Figure 1: Registration flowchart during the RCT period (2019 – 2021).

13b-i) Attrition diagram

Strongly recommended: An attrition diagram (e.g., proportion of participants still logging in or using the intervention/comparator in each group plotted over time, similar to a survival curve) or other figures or tables demonstrating usage/dose/engagement.

subitem not at all important

1 ○

2 ○

3 ○

4 ○

5 ○

essential

Does your paper address subitem 13b-i?

Copy and paste relevant sections from the manuscript or cite the figure number if applicable (include quotes in quotation marks "like this" to indicate direct quotes from your manuscript), or elaborate on this item by providing additional information not in the ms, or briefly explain why the item is not applicable/relevant for your study

Yes: "During the RCT inclusion period, 239 out of 502 women completed the registration they started and 198 women were included in the RCT. Four participants actively dropped out during their follow-up trajectory (control N=1; intervention N=3), however attrition on follow-up questionnaires was much higher (Figure 1)." And see Figure 1: Registration flowchart during the RCT period (2019 – 2021). And see Table 3. ANCOVA and GEE for self-efficacy and the secondary outcomes for response rates on M0 and M6 questionnaires.

14a) Dates defining the periods of recruitment and follow-up

Does your paper address CONSORT subitem 14a? *

Copy and paste relevant sections from the manuscript (include quotes in quotation marks "like this" to indicate direct quotes from your manuscript), or elaborate on this item by providing additional information not in the ms, or briefly explain why the item is not applicable/relevant for your study

Yes: "The outcomes were assessed with online self-report questionnaires at the registration process (M0), at three (M3), six (M6), and 12 months (M12) (Multimedia Appendix 1; source: [32]). The participants for the RCT were largely recruited online, between April 1st 2019 and October 1st 2020 ..."

14a-i) Indicate if critical “secular events” fell into the study period

Indicate if critical “secular events” fell into the study period, e.g., significant changes in Internet resources available or “changes in computer hardware or Internet delivery resources”

subitem not at all important

1 ○

2 ○

3 ○

4 ○

5 ○

essential

Does your paper address subitem 14a-i?

Copy and paste relevant sections from the manuscript (include quotes in quotation marks "like this" to indicate direct quotes from your manuscript), or elaborate on this item by providing additional information not in the ms, or briefly explain why the item is not applicable/relevant for your study

Not applicable in terms of changes in Internet resources available but we did discuss the possible influence of the COVID-19 pandemic on our study: "Furthermore, lockdowns and mitigation measures during the COVID-19 pandemic [10], that occurred simultaneously with a part of this study, not only exacerbated IPVA but also heightened the barriers for disclosing and help seeking [11-14]. ... While other studies found (significant) improvements for depression over time for both study arms [25-27,50] or for a subgroup at three months [23], we did not find a significant effect. This might be related to external circumstances: part of this study took place during the COVID-19 pandemic during which a global increase in depression was observed, especially in women [51-54]. ... With regard to integration [37], we noticed a possible influence of the COVID-19 pandemic and increased attention for DVA and help options during the pandemic’s first wave with an increase in registrations, especially in the first months with restrictions during a national lockdown. ... Furthermore, as the study partially took place during the COVID-19 pandemic, this external circumstance could have decreased or delayed progress or improvement in some outcomes, for example with regard to increasing mental health problems [52,53,66] and a rise in IPVA prevalence and / or severity as well as a diminished access to support services [14,20,67-72]."

14b) Why the trial ended or was stopped (early)

Does your paper address CONSORT subitem 14b? *

Copy and paste relevant sections from the manuscript (include quotes in quotation marks "like this" to indicate direct quotes from your manuscript), or elaborate on this item by providing additional information not in the ms, or briefly explain why the item is not applicable/relevant for your study

Not applicable, the trial was not ended or stopped early.

15) A table showing baseline demographic and clinical characteristics for each group

NPT: When applicable, a description of care providers (case volume, qualification, expertise, etc.) and centers (volume) in each group

Does your paper address CONSORT subitem 15? *

Copy and paste relevant sections from the manuscript (include quotes in quotation marks "like this" to indicate direct quotes from your manuscript), or elaborate on this item by providing additional information not in the ms, or briefly explain why the item is not applicable/relevant for your study

Yes: Table 2. Demographics and scores of the RCT group at baseline (M0; N=198).

15-i) Report demographics associated with digital divide issues

In ehealth trials it is particularly important to report demographics associated with digital divide issues, such as age, education, gender, social-economic status, computer/Internet/ehealth literacy of the participants, if known.

subitem not at all important

1 ○

2 ○

3 ○

4 ○

5 ○

essential

Does your paper address subitem 15-i? *

Copy and paste relevant sections from the manuscript (include quotes in quotation marks "like this" to indicate direct quotes from your manuscript), or elaborate on this item by providing additional information not in the ms, or briefly explain why the item is not applicable/relevant for your study

Yes: Table 2. Demographics and scores of the RCT group at baseline (M0; N=198).

16) For each group, number of participants (denominator) included in each analysis and whether the analysis was by original assigned groups

16-i) Report multiple “denominators” and provide definitions

Report multiple “denominators” and provide definitions: Report N’s (and effect sizes) “across a range of study participation [and use] thresholds” [1], e.g., N exposed, N consented, N used more than x times, N used more than y weeks, N participants “used” the intervention/comparator at specific pre-defined time points of interest (in absolute and relative numbers per group). Always clearly define “use” of the intervention.

subitem not at all important

1 ◯

2 ◯

3 ◯

4 ◯

5 ◯

essential

Does your paper address subitem 16-i? *

Copy and paste relevant sections from the manuscript (include quotes in quotation marks "like this" to indicate direct quotes from your manuscript), or elaborate on this item by providing additional information not in the ms, or briefly explain why the item is not applicable/relevant for your study

Yes: "During the RCT inclusion period, 239 out of 502 women completed the registration they started and 198 women were included in the RCT. Four participants actively dropped out during their follow-up trajectory (control N=1; intervention N=3), however attrition on follow-up questionnaires was much higher (Figure 1)." See Figure 1. Registration flowchart during the RCT period (2019 – 2021) and Multimedia Appendix 3. User data from the RCT study arms and OFS group.

16-ii) Primary analysis should be intent-to-treat

Primary analysis should be intent-to-treat, secondary analyses could include comparing only “users”, with the appropriate caveats that this is no longer a randomized sample (see 18-i).

subitem not at all important

1 ○

2 ○

3 ○

4 ○

5 ○

essential

Does your paper address subitem 16-ii?

Copy and paste relevant sections from the manuscript (include quotes in quotation marks "like this" to indicate direct quotes from your manuscript), or elaborate on this item by providing additional information not in the ms, or briefly explain why the item is not applicable/relevant for your study

Yes: "The data was analyzed with descriptive statistics (based on intention to treat), ANCOVAs, and Generalized Estimated Equations (GEEs) for the primary and secondary outcomes, controlling for baseline scores and using SPSS, version 25 [35] (Table 3). For self-efficacy, a complete case analysis (CCA) was conducted as well for M0, M3 and M6."

17a) For each primary and secondary outcome, results for each group, and the estimated effect size and its precision (such as 95% confidence interval)

Does your paper address CONSORT subitem 17a? *

Copy and paste relevant sections from the manuscript (include quotes in quotation marks "like this" to indicate direct quotes from your manuscript), or elaborate on this item by providing additional information not in the ms, or briefly explain why the item is not applicable/relevant for your study

Yes, if applicable: "We could not detect any statistically significant differences in the primary outcome of self-efficacy between the study arms in the ANCOVA (M6: p=.850), GEE (p=.976) (Table 3) and CCA (p=.856)." See: Table 3. ANCOVA and GEE for self-efficacy and the secondary outcomes.

17a-i) Presentation of process outcomes such as metrics of use and intensity of use

In addition to primary/secondary (clinical) outcomes, the presentation of process outcomes such as metrics of use and intensity of use (dose, exposure) and their operational definitions is critical. This does not only refer to metrics of attrition (13-b) (often a binary variable), but also to more continuous exposure metrics such as “average session length”. These must be accompanied by a technical description how a metric like a “session” is defined (e.g., timeout after idle time) [1] (report under item 6a).

subitem not at all important

1 ○

2 ○

3 ○

4 ○

5 ○

essential

Does your paper address subitem 17a-i?

Copy and paste relevant sections from the manuscript (include quotes in quotation marks "like this" to indicate direct quotes from your manuscript), or elaborate on this item by providing additional information not in the ms, or briefly explain why the item is not applicable/relevant for your study

Yes: "The RCT user data showed that 22 participants in the intervention group and 26 participants in the control group never logged in (Multimedia Appendix 3). The intervention group spent significantly more time on the intervention than the control group (p<.01) but we found no significant difference between the study arms for the average amount of logins (p=.078). Women in the RCT intervention group and OFS mostly visited the interactive contact options, such as the forum. The RCT control group mostly used the pages on help options (Multimedia Appendix 3)."

17b) For binary outcomes, presentation of both absolute and relative effect sizes is recommended

Does your paper address CONSORT subitem 17b? *

Copy and paste relevant sections from the manuscript (include quotes in quotation marks "like this" to indicate direct quotes from your manuscript), or elaborate on this item by providing additional information not in the ms, or briefly explain why the item is not applicable/relevant for your study

Not applicable for this study.

18) Results of any other analyses performed, including subgroup analyses and adjusted analyses, distinguishing pre-specified from exploratory

Does your paper address CONSORT subitem 18? *

Copy and paste relevant sections from the manuscript (include quotes in quotation marks "like this" to indicate direct quotes from your manuscript), or elaborate on this item by providing additional information not in the ms, or briefly explain why the item is not applicable/relevant for your study

Yes: "In Europe, our team in the Netherlands developed the first eHealth intervention for female IPVA survivors that is scientifically evaluated through a randomized controlled trial (RCT), a process evaluation (PE), and an open feasibility study (OFS). ... This article focuses on two main outcomes derived from the RCT, quantitative PE, and OFS: effectiveness and feasibility. ... We also conducted a qualitative PE (interviews), described in a separate article [36]." Thus, the outcomes of the quantitative process evaluation and the open feasibility study can be also be found in this article. The outcomes of the qualitative evaluation are described in a separate article.

18-i) Subgroup analysis of comparing only users

A subgroup analysis of comparing only users is not uncommon in ehealth trials, but if done, it must be stressed that this is a self-selected sample and no longer an unbiased sample from a randomized trial (see 16-iii).

subitem not at all important

1 ○

2 ○

3 ○

4 ○

5 ○

essential

Does your paper address subitem 18-i?

Copy and paste relevant sections from the manuscript (include quotes in quotation marks "like this" to indicate direct quotes from your manuscript), or elaborate on this item by providing additional information not in the ms, or briefly explain why the item is not applicable/relevant for your study

Not applicable to this study, we did not conduct this type of analysis.

19) All important harms or unintended effects in each group
(for specific guidance see CONSORT for harms)

Does your paper address CONSORT subitem 19? *

Copy and paste relevant sections from the manuscript (include quotes in quotation marks "like this" to indicate direct quotes from your manuscript), or elaborate on this item by providing additional information not in the ms, or briefly explain why the item is not applicable/relevant for your study

Not applicable: "Taken together, the intervention's self-help nature, its extensive reach within society (Multimedia Appendix 3), and no evidence of harmful effects, make this intervention very sustainable and easy to integrate in existing care and support structures [26,37]."

19-i) Include privacy breaches, technical problems

Include privacy breaches, technical problems. This does not only include physical "harm" to participants, but also incidents such as perceived or real privacy breaches [1], technical problems, and other unexpected/unintended incidents. "Unintended effects" also includes unintended positive effects [2].

subitem not at all important

1 ○

2 ○

3 ○

4 ○

5 ○

essential

Does your paper address subitem 19-i?

Copy and paste relevant sections from the manuscript (include quotes in quotation marks "like this" to indicate direct quotes from your manuscript), or elaborate on this item by providing additional information not in the ms, or briefly explain why the item is not applicable/relevant for your study

Privacy breaches did not occur. Technical problems are assessed with the Web Evaluation Questionnaire, see Table 4. WEQ outcomes for RCT study arms. Additional information on for example login problems reported by the participants can be found in the qualitative evaluation that is discussed in a separate article (under review).

19-ii) Include qualitative feedback from participants or observations from staff/researchers

Include qualitative feedback from participants or observations from staff/researchers, if available, on strengths and shortcomings of the application, especially if they point to unintended/unexpected effects or uses. This includes (if available) reasons for why people did or did not use the application as intended by the developers.

subitem not at all important

1 ○

2 ○

3 ○

4 ○

5 ○

essential

Does your paper address subitem 19-ii?

Copy and paste relevant sections from the manuscript (include quotes in quotation marks "like this" to indicate direct quotes from your manuscript), or elaborate on this item by providing additional information not in the ms, or briefly explain why the item is not applicable/relevant for your study

This is described in a separate article on the qualitative evaluation of this intervention.

DISCUSSION

22) Interpretation consistent with results, balancing benefits and harms, and considering other relevant evidence

NPT: In addition, take into account the choice of the comparator, lack of or partial blinding, and unequal expertise of care providers or centers in each group

22-i) Restate study questions and summarize the answers suggested by the data, starting with primary outcomes and process outcomes (use)

Restate study questions and summarize the answers suggested by the data, starting with primary outcomes and process outcomes (use).

subitem not at all important

1 ○

2 ○

3 ○

4 ○

5 ○

essential

Does your paper address subitem 22-i? *

Copy and paste relevant sections from the manuscript (include quotes in quotation marks "like this" to indicate direct quotes from your manuscript), or elaborate on this item by providing additional information not in the ms, or briefly explain why the item is not applicable/relevant for your study

Yes, for example: "This study quantitatively evaluated the effectiveness and feasibility of the first Dutch eHealth intervention for women exposed to IPVA: SAFE. This study did not provide statistically significant evidence that the extensive SAFE intervention was more effective than the minimal intervention in increasing self-efficacy (primary outcome), awareness and perceived support, and in decreasing mental health problems (secondary outcomes). It did provide evidence for SAFE's adequate feasibility on multiple levels, such as acceptability and demand, and for participants' satisfaction and appreciation."

22-ii) Highlight unanswered new questions, suggest future research

Highlight unanswered new questions, suggest future research.

subitem not at all important

1 ○

2 ○

3 ○

4 ○

5 ○

essential

Does your paper address subitem 22-ii?

Copy and paste relevant sections from the manuscript (include quotes in quotation marks "like this" to indicate direct quotes from your manuscript), or elaborate on this item by providing additional information not in the ms, or briefly explain why the item is not applicable/relevant for your study

Yes: "Overall, we might have to reconsider our expectations for online interventions since the ones specifically designed to treat symptoms of anxiety and depression only yield small effects for their target populations. Nevertheless, they can be helpful and meaningful for health outcomes in the general population [77,78]. Most importantly, IPVA survivors may not seek online support for this purpose. Thus, we might have to rethink how we design and evaluate these interventions. The RCT might not be the most suitable evaluation method in this context [79,80]. Instead, actively including the target group in designing the intervention and employing multiple methods of evaluation, quantitative and qualitative, appears crucial towards obtaining real world, in-depth knowledge about the effectiveness of an eHealth intervention for IPVA survivors [31,36]. Last, in both design and research, interventions should pay attention to diversity on multiple levels, for example with regard to cultural sensitivity and availability in multiple languages [36,81-83]."

20) Trial limitations, addressing sources of potential bias, imprecision, and, if relevant, multiplicity of analyses

20-i) Typical limitations in ehealth trials

Typical limitations in ehealth trials: Participants in ehealth trials are rarely blinded. Ehealth trials often look at a multiplicity of outcomes, increasing risk for a Type I error. Discuss biases due to non-use of the intervention/usability issues, biases through informed consent procedures, unexpected events.

subitem not at all important

1 ○

2 ○

3 ○

4 ○

5 ○

essential

Does your paper address subitem 20-i? *

Copy and paste relevant sections from the manuscript (include quotes in quotation marks "like this" to indicate direct quotes from your manuscript), or elaborate on this item by providing additional information not in the ms, or briefly explain why the item is not applicable/relevant for your study

Yes, for example: "There are also several limitations to this study. First, we noticed the extensive registration procedure for the RCT study was a barrier for women to sign up for the intervention. Also, unlike other studies [25-27], we encountered a high attrition rate with regard to responses on follow-up questionnaires, leading to a small sample size and possibly a power problem (for example with social support (MOS-SS5) at M6, see Table 3). With regard to attrition, we found signs of selective attrition bias for self-efficacy and sexual IPVA that may have influenced the outcomes. ... Second, the reliance on self-reports for all outcomes and thus the risk of self-reporting bias is a limitation. Furthermore, as the study partially took place during the COVID-19 pandemic, this external circumstance could have decreased or delayed progress or improvement in some outcomes, for example with regard to increasing mental health problems [52,53,66] and a rise in IPVA prevalence and / or severity as well as a diminished access to support services [14,20,67-72]."

21) Generalisability (external validity, applicability) of the trial findings

NPT: External validity of the trial findings according to the intervention, comparators, patients, and care providers or centers involved in the trial

21-i) Generalizability to other populations

Generalizability to other populations: In particular, discuss generalizability to a general Internet population, outside of a RCT setting, and general patient population, including applicability of the study results for other organizations

subitem not at all important

1 ○

2 ○

3 ○

4 ○

5 ○

essential

Does your paper address subitem 21-i?

Copy and paste relevant sections from the manuscript (include quotes in quotation marks "like this" to indicate direct quotes from your manuscript), or elaborate on this item by providing additional information not in the ms, or briefly explain why the item is not applicable/relevant for your study

Yes: Last, in terms of diversity and equity, this study has some limitations as well [14,73]. The intervention was only available in Dutch which excluded women who did not sufficiently comprehend Dutch. Also, while many people in the Netherlands have access to the internet (97%) and are digitally literate (50% has ‘above basic overall digital skills’) [74,75], women who did not have access to the internet or who do not know how to use it were excluded. With regard to educational diversity, a noticeably higher percentage of the sample (50,5%) has a high education level compared to the general female population in the Netherlands (34%) [76]."

21-ii) Discuss if there were elements in the RCT that would be different in a routine application setting

Discuss if there were elements in the RCT that would be different in a routine application setting (e.g., prompts/reminders, more human involvement, training sessions or other co-interventions) and what impact the omission of these elements could have on use, adoption, or outcomes if the intervention is applied outside of a RCT setting.

subitem not at all important

1 ○

2 ○

3 ○

4 ○

5 ○

essential

Does your paper address subitem 21-ii?

Copy and paste relevant sections from the manuscript (include quotes in quotation marks "like this" to indicate direct quotes from your manuscript), or elaborate on this item by providing additional information not in the ms, or briefly explain why the item is not applicable/relevant for your study

Yes, we conducted an open feasibility study for this purpose. For example: "For the OFS, the complete version of the intervention (Table 1) was available without an extensive mandatory registration procedure and baseline questionnaire. Women accessed the intervention with a nickname and self-selected password. For access to the forum, women had to answer a few questions about for example their gender identity and age (Multimedia Appendix 2) and provide an e-mail address in order to ensure safety for forum users. The chat feature was not offered as initially planned, given the rapid growth in users and the inability to monitor the chat 24/7 by the community managers, and the relatively low active use. Hence, we decided to remove this option, this is a change to protocol, as a preventative measure to guarantee user safety at all times. ... We saw an increase of registrations during the OFS (N=170; RCT and OFS means per month are respectively 13,2 and 56,7; Figure 2). ... Also, we noticed an increase in registrations during the OFS, which was expected due to the change from an extensive registration process during the RCT to an easy, direct access registration during the OFS. ... First, we noticed the extensive registration procedure for the RCT study was a barrier for women to sign up for the intervention."

OTHER INFORMATION

23) Registration number and name of trial registry

Does your paper address CONSORT subitem 23? *

Copy and paste relevant sections from the manuscript (include quotes in quotation marks "like this" to indicate direct quotes from your manuscript), or elaborate on this item by providing additional information not in the ms, or briefly explain why the item is not applicable/relevant for your study

Yes: "Registration: Netherlands Trial Register NL7108 (NTR7313; https://trialsearch.who.int/Trial2.aspx?TrialID=NTR7313)."

24) Where the full trial protocol can be accessed, if available

Does your paper address CONSORT subitem 24? *

Cite a Multimedia Appendix, other reference, or copy and paste relevant sections from the manuscript (include quotes in quotation marks "like this" to indicate direct quotes from your manuscript), or elaborate on this item by providing additional information not in the ms, or briefly explain why the item is not applicable/relevant for your study

Yes, the study protocol was published earlier and can be accessed at: https://bmcpublichealth.biomedcentral.com/articles/10.1186/s12889-020-08743-0

25) Sources of funding and other support (such as supply of drugs), role of funders

Does your paper address CONSORT subitem 25? *

Copy and paste relevant sections from the manuscript (include quotes in quotation marks "like this" to indicate direct quotes from your manuscript), or elaborate on this item by providing additional information not in the ms, or briefly explain why the item is not applicable/relevant for your study

Yes: "This study is government funded by the Gender and Health program of ZonMw (grant number 849200002)."

X27) Conflicts of Interest (not a CONSORT item)

X27-i) State the relation of the study team towards the system being evaluated

In addition to the usual declaration of interests (financial or otherwise), also state the relation of the study team towards the system being evaluated, i.e., state if the authors/evaluators are distinct from or identical with the developers/sponsors of the intervention.

subitem not at all important

1 ○

2 ○

3 ○

4 ○

5 ○

essential

Does your paper address subitem X27-i?

Copy and paste relevant sections from the manuscript (include quotes in quotation marks "like this" to indicate direct quotes from your manuscript), or elaborate on this item by providing additional information not in the ms, or briefly explain why the item is not applicable/relevant for your study

Yes: "Conflicts of Interest: The SAFE intervention that was evaluated in this study was designed by the authors and built by an eHealth developer (Ippo) outside the Radboudumc."

About the CONSORT EHEALTH checklist

As a result of using this checklist, did you make changes in your manuscript? *

- ○ yes, major changes
- ◉ yes, minor changes
- ○ no

What were the most important changes you made as a result of using this checklist?

Providing additional information.

How much time did you spend on going through the checklist INCLUDING making changes in your manuscript *

Filling out the checklist and making changes to the manuscript took several hours.

As a result of using this checklist, do you think your manuscript has improved? *

- (x) yes
- ( ) no
- ( ) Anders:

Would you like to become involved in the CONSORT EHEALTH group?

This would involve for example becoming involved in participating in a workshop and writing an "Explanation and Elaboration" document

- ( ) yes
- ( ) no
- ( ) Anders:

Any other comments or questions on CONSORT EHEALTH

Jouw antwoord

STOP - Save this form as PDF before you click submit

To generate a record that you filled in this form, we recommend to generate a PDF of this page (on a Mac, simply select "print" and then select "print as PDF") before you submit it.

When you submit your (revised) paper to JMIR, please upload the PDF as supplementary file.

Don't worry if some text in the textboxes is cut off, as we still have the complete information in our database. Thank you!

Final step: Click submit !

Click submit so we have your answers in our database!

Verzenden Formulier wissen

Verzend nooit wachtwoorden via Google Formulieren.

Deze content is niet gemaakt of goedgekeurd door Google. Misbruik rapporteren - Servicevoorwaarden - Privacybeleid

Google Formulieren
